# Methods women use for induced abortion and sources of services: insights from poor urban settlements of Accra, Ghana

**DOI:** 10.1186/s12905-021-01444-9

**Published:** 2021-08-16

**Authors:** Caesar Agula, Elizabeth G. Henry, Patrick O. Asuming, Charles Agyei-Asabere, Mawuli Kushitor, David Canning, Iqbal Shah, Ayaga A. Bawah

**Affiliations:** 1 Regional Institute for Population Studies (RIPS), University of Ghana, Accra, Ghana; 2 Department of Global Health and Population, Harvard T.H. Chan School of Public Health, Boston, USA; 3 University of Ghana Business School (UGBS), University of Ghana, Accra, Ghana

**Keywords:** Induced abortion, Abortion methods, Source of abortion service, Urban-poor, Ghana

## Abstract

**Background:**

Increasing access to safe abortion methods is crucial for improving women’s health. Understanding patterns of service use is important for identifying areas for improvement. Limited evidence is available in Ghana on factors associated with the type of method used to induce abortion. This paper examined the methods and sources of services used for abortion by women living in poor urban settings of Accra.

**Methods:**

Data are from a survey that was conducted in 2018 among 1233 women aged 16–44 years who reported ever having had an induced abortion. We estimated a multinomial logistic regression model to examine factors associated with the type of abortion methods women used. We further generated descriptive statistics for the source of abortion services.

**Results:**

About 50% women used surgical procedures for their last abortion, 28% used medication abortion (MA), 12% used other pills, 3% used injection, and 7% used non-medical methods. However, nearly half (46%) of the women who terminated a pregnancy within the year preceding the survey used medication abortion (MA), 32% used surgical procedures, while 5% used non-medical methods. Women who terminated a pregnancy within three years preceding the survey had a 60% lower chance of using surgical procedures if they did not use MA compared to those who terminated a pregnancy more than 3 years before the survey (Relative Risk Ratio [RRR] 0.4; 95% CI 0.3–0.5). The vast majority (74%) of women who used MA obtained services from pharmacies.

**Conclusions:**

The use of MA pills to terminate pregnancies has increased in recent years in Ghana and these pills are mostly accessed from pharmacies. This suggests a need for a review of the national guidelines to include pharmacists and chemists in the provision of MA services.

**Supplementary Information:**

The online version contains supplementary material available at 10.1186/s12905-021-01444-9.

## Background

Each year an estimated 56 million induced abortions occur worldwide and nearly 45% of these are unsafe [1]. According to the World Health Organization (WHO), abortion is unsafe when the pregnancy is terminated by a person lacking the required medical skills or performed in an environment that does not meet the minimal medical standards, or both [2, 3]. Depending on the type of abortion method (i.e. through medication pills or surgical methods) and the gestational age of the pregnancy, the person, skills and medical standards considered safe may vary [2]. Therefore, WHO has further categorized unsafe abortion into: less-safe and least-safe [4]. “Less-safe” abortions are those done by trained providers using non-recommended methods or using a safe method (for example, misoprostol) but without adequate information or support from a trained individual and “least-safe” abortions are those done by untrained people using dangerous, invasive methods [4]. An estimated 97% of unsafe abortions occur in the developing world [4]. Africa has the highest fatality rate for unsafe induced abortion, with 460 deaths per 100,000 unsafe abortions according to 2008 estimates [5]. In addition, almost half of all deaths related to unsafe abortion worldwide occur in sub-Saharan Africa [6].

Most countries in sub-Saharan Africa and Africa at large have restrictive abortion laws [7]. The abortion law in Ghana, inherited from the United Kingdom colonial government was also initially restrictive, as abortion was criminalized in the penal code [8]. Overtime, and with decreasing influence of institutionalized religion, there was pressure for abortion to be liberalized in Ghana [8]. In 1985, Ghana amended the abortion law to allow for legal termination of pregnancy when there is evidence of rape, incest or “defilement of an idiot”, risk of fetal impairment and/or if the pregnancy would affect her physical or mental health [9]. Despite what is often considered a relatively liberal abortion law, close to half (≈ 45%) of abortions in Ghana are estimated to be performed in unsafe conditions [9]. Abortion in Ghana accounts for an estimated 15–30% of all maternal deaths, making it a leading contributor to maternal mortality in the country [6]. In addition to the fatalities, many women suffer non-fatal health complications from unsafe abortions [10].

Some of the main barriers to accessing safe abortion services in developing settings like Ghana include stigma and inadequate knowledge of the legal framework for safe termination of pregnancy [11]. According to Aniteye and colleagues, women seeking safe abortion in Ghana sometimes experience stigma from both health providers and society, and this often leads to self-inducing abortions and the use of methods that are unsafe [11]. In addition, insufficient knowledge of abortion law may lead to the use of unsafe methods to terminate pregnancies [11, 12]. For instance, qualitative work in Ashanti region showed that women who perceived abortion as illegal terminated pregnancy using unsafe methods [12].

Access to safe abortion methods from trained providers is crucial for the provision of high quality comprehensive sexual and reproductive health care [13]. Such methods include surgical procedures (manual vacuum aspiration (MVA), electric vacuum aspiration (EVA) and dilation and evacuation (D&E)) and medication (misoprostol-only tablets and mifepristone with misoprostol combination tablets) [2]. With insufficient trained service providers [14], especially in countries where abortion is restricted to specialized providers in licensed health facilities, expanding safe abortion methods that do not rely on providers who require surgical training is one strategy to improve access [15]. Like surgical procedures in health facilities, self-managed abortion using medication abortion (MA) pills has been proven to be safe and effective, and thus play an important role in the provision of safe abortion particularly in several contexts [16]. A recent study in Nepal showed that non-physicians such as trained pharmacy service providers can provide safe and effective medication abortion [17].

The Ministry of Health (MoH), Ghana, included mifepristone in the list of essential medicines in the country in 2010, which was an addition to misoprostol that had been included in the list in 2004 [18]. Data suggest that a high proportion of women are accessing MA. In 2017, among women aged 15–49 years who ever had induced abortion, 31% reported using surgical methods for their last abortion, 38% used MA, 27% used non-medical methods including the use of herbs, homemade concoctions/products, insertion of objects/leaves, heavy massage or unknown types of tablets and 3% reported utilizing injection and other methods [19]. Before the introduction of MA in Ghana by Marie Stopes International (in 2015), safe induced abortion was restricted to surgical procedures performed by health professionals specialized in gynecology or registered health practitioners in specific abortion licensed health facilities [11]. Though effort has been made through special initiatives to increase the availability of MA in licensed franchised facilities including some pharmacies, the reach is limited. At the same time, unregistered products for terminating pregnancies have also become widely available at pharmacies. It is unclear who is accessing each type of method to induce abortion, and from where.

There is no documentation of the characteristics of women who use the different methods for terminating pregnancies in Ghana. Some research from Northwest Ethiopia shows that women are more likely to choose MA compared with surgical methods if they live in urban areas, and early in the pregnancy, which is consistent with WHO guidelines [20]. Studies conducted in Ghana found that older age, having legal knowledge of abortion law and financial support were some of the factors associated with the use of safe abortion services [21–23]. Findings from the Ghana Maternal Health Survey (GMHS) show that MA is used more by younger than older women [19]. However, the demographic and socio-economic background characteristics of women using specific abortion methods have not been explored, especially among poor urban communities. In addition, little is known about where or from whom women in poor urban communities who need abortion services obtain them. With increased migration of rural populations to cities in Ghana, it is important to understand the reproductive health needs of poor urban women who are usually vulnerable to the adverse health effects of urbanization and often lack access to health services and resources [24–26].

Our study addresses these gaps by examining the factors associated with the type of method used to terminate pregnancies, as well as the source of abortion method. The paper hypothesizes that socio-economic characteristics and abortion-elated attributes such as knowledge of abortion law and the duration since the introduction of MA in Ghana are associated with the type of method women used to terminate pregnancies. Understanding the types and sources of method used by women in poor urban settings to terminate pregnancies is important for informing strategies to improve access to safe abortion services in particular and maternal and reproductive health outcomes in general.

## Methods

### Study setting and sampling

Data used in this paper are from the Willows Impact Evaluation project implemented in Ghana (hereafter, referred to as “WIE-Ghana) between 2017 and 2018. WIE-Ghana collected reproductive health information from women living in two urban-poor neighborhoods of Accra, the capital city of Ghana, West Africa. The two study settings were purposively identified on the basis of having similar demographic, ethnic and socio-economic characteristics. One study setting was in the coastal area while the other was located approximately 18 km inland. The communities surveyed in the coastal area included Osu Klotey, La, Teshie and Nungua while those surveyed in the inland area were La Nkwantanang (Madina), Abogba and Old Ashongman. These communities are disproportionately vulnerable to shocks including floods, congestion, water scarcity, sanitation problems, cholera and other health hazards [24–26].

**Fig. 1:**
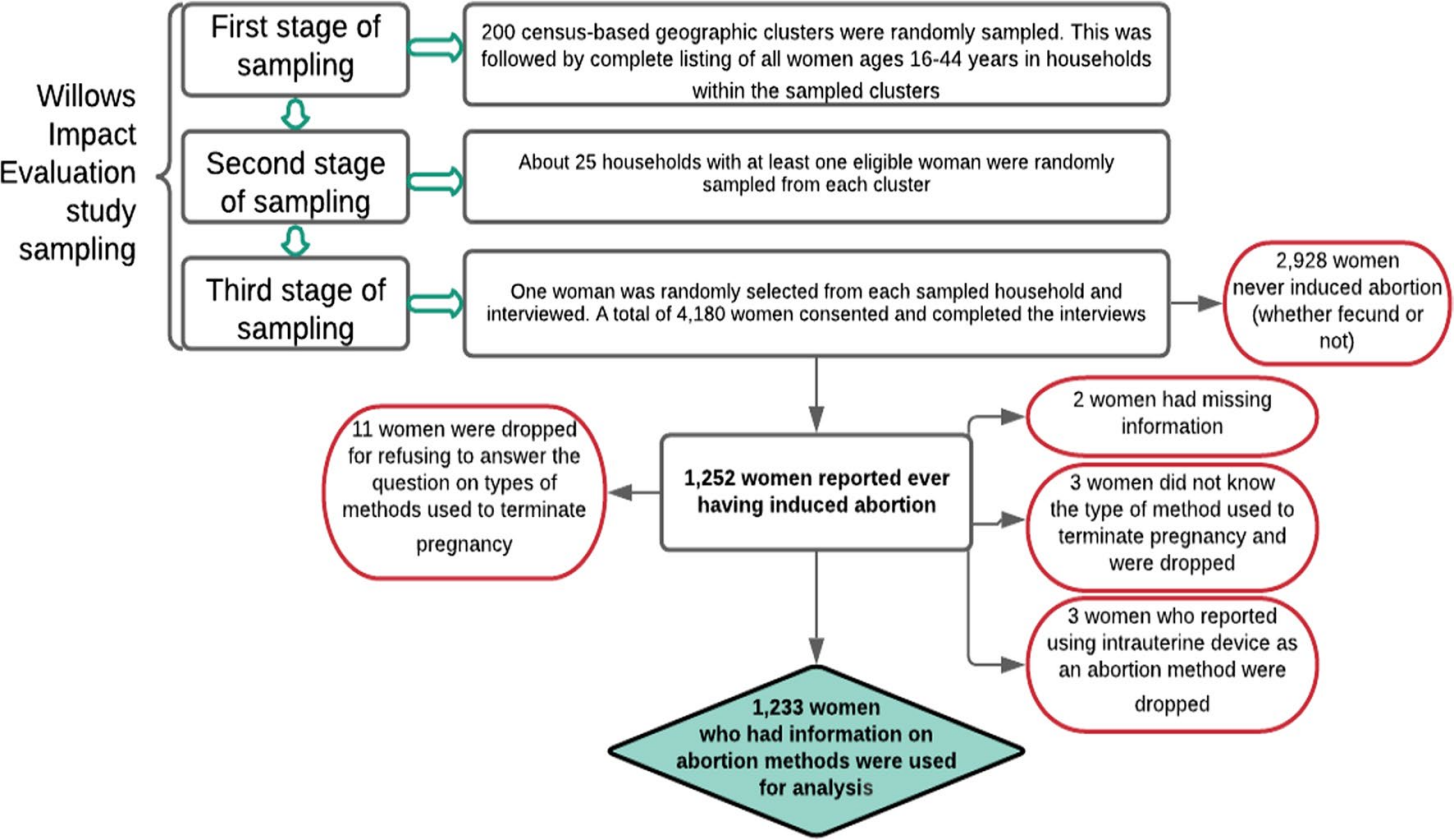
Sampling and procedure for obtaining final analytic sample. *Source*: Constructed by authors

WIE-Ghana employed a three-stage cluster sampling technique to create representative samples from each of the two study settings (Fig. 1). In the first stage, a simple random sampling technique was used to sample 200 census-based geographic clusters (100 in each for the coastal and inland areas). The clusters were generally equally-sized subdivisions of census Enumeration Areas (EAs) obtained from the Ghana Statistical Service consisting of approximately 60–100 households. A complete listing of all eligible women ages 16–44 years in households within the sampled clusters was conducted. In the second stage, approximately 25 households with at least one eligible woman were randomly sampled from each cluster. In the final stage, if the sampled households had more than one eligible woman, one woman was randomly selected for the interview. Our final analytic sample for this paper included 1252 women (representing 30% of WIE-Ghana sample) who reported ever having had an induced abortion. Of these, eleven refused to answer the question on type of method, three did not know, and two had missing information, leaving 1236 women. Three other women who indicated the use of IUD as an abortion method were dropped during data cleaning, leaving 1233 women for analysis.

### Data collection

Data were collected between January and July, 2018. Data collection was conducted using questionnaires programmed in CommCare version 2.40.1 for use in tablets [27]. Face-to-face interviews were conducted by trained research assistants in either English or the local language after obtaining written consent. The interviews took place in or within the vicinity of the respondents’ homes or places of work. The interviews captured household, demographic, socio-economic, and sexual and reproductive health information, including abortion (Additional file 1). Given the sensitive nature of some of the topics, research assistants were extensively trained by the supervising team from the Regional Institute for Population Studies at the University of Ghana, the Harvard T.H. Chan School of Public Health and Ghana Health Service on interviewing techniques, and topics such as abortion law and methods for abortion.

### Measures

The questionnaire had a series of questions on abortion for women who reported that they have ever had a pregnancy that ended in miscarriage, induced abortion or stillbirth (Additional file 1). Women who reported that they had ever intentionally terminated a pregnancy or induced an abortion were further asked about the number of pregnancies that ended in induced abortion, when the last induced abortion occurred, and what was done to have their recent pregnancy terminated. To capture information on methods used to induce abortion, women were asked, “What was done to have the pregnancy terminated?”. Response categories included: surgical (operation), injection, took pills/medicines/medication abortion, inserted herbs or object in womb, took homemade medicine and other (specify). Women who responded that they used a surgical method were further asked, “What was the name of the surgical procedure used to end pregnancy?”. Response categories include: MVA or EVA, Dilation and Curettage (D&C)/Sharp Curettage and specify other. Women who responded that they took pills/medicines or medication abortion were, on the other hand, asked, “What kind of medication did you use to end the pregnancy?”. Response categories included: Mifepristone and Misoprostol (Medabon/Mariprist), Misoprostol alone (Cytotec/Misoclear), Oral contraceptive pills (OCP) (Lydia, Microgynon), Other pills and Don’t know, and Other (Specify). If women mentioned either the brand-name or the actual medication, these options were recorded under appropriate response options. However, the women were not prompted and if they did not recall the name or brand “Other pills” was selected. These questions collectively formed the basis for categorizing types of abortion methods in this study.

For source of service for the last induced abortion, women were asked, “From whom/where did you receive induced abortion services the last time?”. There were 21 possible response categories that were ultimately compiled into the following: government hospital/polyclinic, government health center/clinic, government health post/CHPS, private hospital/clinic, private doctor, pharmacy/chemical/drug stores, friends/relative/partner, drug peddler and other (specify).

Other explanatory variables included: age of woman (16–19 years, 20–24 years, 25–29 years, 30–34 years, 35–39 years and 40–44 years); marital status (never in union, currently in union and formerly in union); level of education (no formal education, completed primary, completed middle school/junior high school (JHS), completed secondary and higher); religion (Moslem, Catholic, Anglican/Methodist/Presbyterian, Pentecostal, other Christian and other/no religion); ethnicity (Ga-Dangme, Akan, Ewe, other Ghanaian and non-Ghanaian); household wealth index (poorest, poorer, middle, richer and richest); knowledge of abortion law (knowledge of at least one legal condition to induce abortion and no knowledge of any legal condition in Ghana); and when last induced abortion was conducted (induced abortion more than 3 years ago and induced abortion recently within 3 years).

### Analysis

Data from CommCare was exported to STATA version 14.2 for analysis [28]. Statistical tests with p-values (p < 0.05) were considered significant. We generated descriptive statistics for socio-economic, demographic and abortion-related attributes, including sources of services. We then estimated a multinomial logistic regression model to examine the factors associated with the type of method used for the last induced abortion. For the regression modelling, we classified the types of abortion methods into three broad categories: Surgical (D&C and EVA/MVA), Medication (misoprostol alone and misoprostol and mifepristone combination) and Non-medical methods (insertion of objects, and homemade herbs/concoctions/objects). Medication abortion was used as the base outcome for the analysis. Women who reported using other pills (approximately 12%) were dropped from the analysis since the use of specific medication abortion pills may be significantly different from the use of OCP or other non-recommended pharmaceutical tablets. However, it is also possible that women used approved MA pills but did not remember the brand name. A sensitivity analysis was therefore conducted by including OCP and other non-recommended pharmaceutical tablets to the medication category. We also dropped women who reported using injection (about 3%) from the regression analysis. Injection abortion (methotrexate alone or combination of methotrexate and misoprostol) uses similar regimens as medication abortion. However, it could not be classified under MA because the medium of use is significantly different and is often used by doctors for rare abortion cases such as ectopic pregnancy and for women who are allergic to mifepristone [29, 30]. The explanatory variables included in the regression analysis were selected based on the existing literature and their likely role in influencing choice of abortion methods [20, 31, 32]. The results are presented as relative risk ratios (RRR) with 95% confidence intervals (CI).

### Ethical considerations

Ethical approvals were obtained from Ghana Health Service Ethical Review Committee (GHS-ERC #: 005/08/2017), University of Ghana Ethics Committee for the Humanities (ECH #: 020/17–18) and the Institutional Review Board (IRB) of Harvard T.H. Chan School of Public Health before implementing the study. Written informed consent was obtained for all respondents before participation in the study. Before obtaining the written informed consent, an information sheet which contained a summary of the study and all ethical issues related to the study was given to the participant to read. For participants with no formal education, the research assistants read and explained the information sheet to them. Since data were collected from women aged 16 to 44 years, no parental/guardian consent was required. In Ghana, minors are persons under the age of 16 years.

## Results

### Background characteristics of women

Table 1 presents the socio-economic, demographic and abortion-related attributes of the respondents. The majority (62%) of women were married or in union at the time of the survey while the highest proportion (27%) were aged 30–34 years. Twenty-five percent of the women had secondary or higher levels of education while 16% had no formal education. Most (59%) of the women belonged to Pentecostal/charismatic Christian religion while only 5% were Moslems. The Ga-Dangme ethnic group, being the indigenous people of Accra, were the largest ethnic group in the sample (38%), followed by the Akans (35%). These two ethnic groups accounted for the majority of women (73%) included in the study. About one-tenth (11%) of the women were from households in the lowest wealth quintile while 4% were from households in the highest wealth quintile.

**Table 1:** Socio-economic, demographic and abortion-related attributes of respondents

Variable	Cases (n = 1233)	Percent (%)
*Woman's age*		
16–19	16	1.3
20–24	136	11.0
25–29	271	22.0
30–34	337	27.3
35–39	273	22.1
40–44	200	16.2
*Marital status*		
Never in union	317	25.7
Currently in union	768	62.3
Formerly in union	147	11.9
*Level of education*		
No formal education	199	16.3
Completed primary	218	17.8
Completed middle/junior high school	497	40.6
Completed secondary	240	19.6
Higher	70	5.7
*Religion*		
Moslem	63	5.1
Catholic	27	2.2
Anglican/Methodist/Presbyterian	143	11.6
Pentecostal	731	59.3
Other Christian	239	19.4
Other/no religion	30	2.4
*Ethnicity*		
Ga/Dangme	468	38.0
Akan	436	35.4
Ewe	220	17.9
Other Ghanaian	95	7.7
Non Ghanaian	13	1.1
*Wealth index*^*Ψ*^		
Poorest	138	11.2
Poorer	290	23.5
Middle	442	35.9
Richer	318	25.8
Richest	45	3.7
*Knowledge of abortion law*		
Knowledge of at least one legal condition to induce abortion	221	17.9
No knowledge of any legal conditions	1012	82.1
*Year since last induced abortion*		
< = 3	411	34.1
> 3	795	65.9
*Abortion methods*		
Surgical	620	50.3
Dilation and curettage/dilation and evacuation	393	31.9
Manual vacuum aspiration	132	10.7
Surgical-Don’t know	95	7.7
Medication	341	27.7
Mifepristone + Misopristol	133	10.8
Misopristol	208	16.9
Other pills (oral contraceptive pills, unnamed)	149	12.1
Injection	39	3.2
Non-medical	84	6.8
Inserted objects	14	1.1
Homemade/herbal	70	5.7

^Ψ^Wealth index was computed from household assets and amenities using principal component analysis

Regarding abortion-related attributes, there was generally poor knowledge about the legal grounds under which abortion is permitted in Ghana, with only 18% of the women correctly identifying at least one condition under which abortion is legally permitted in the country (Table 1). Half of all last induced abortions involved surgical procedures, with 32% using D&C or D&E, 11% MVA, while 8% were unknown type of surgical method. Twenty-eight percent of the abortions involved MA pills, with 17% involving misoprostol only while 11% involved a combination of mifepristone and misoprostol. Twelve percent of the abortions involved use of tablets of unknown type, 3% involved injection, and 7% involved non-medical methods. Two-thirds (66%) of the abortions occurred more than three years before the survey.

### Methods used for abortion among urban-poor women

**Fig. 2:**
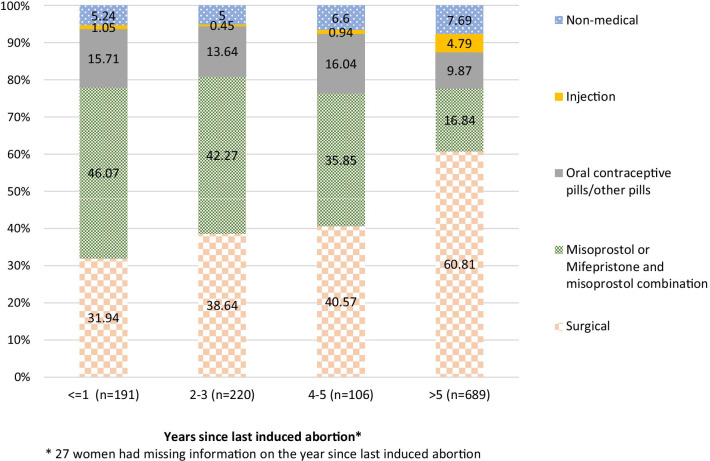
Last induced abortion methods women used by years since last abortion

Figure 2 presents the last induced abortion method that women used by years since last abortion: less than one year ago, 2–3 years ago, 4–5 years ago and more than 5 years from the time of the survey. Among women having the last abortion over five years before the survey, about 60% had abortion by surgical methods. The reliance on surgical methods declined with time to 32% among women who terminated a pregnancy during the year prior to the survey. The proportion of women using MA increased from 17% for women who last terminated a pregnancy more than five years before the survey to 46% for women who terminated within the past year. The proportion of women using non-medical methods declined from 8% of those who terminated a pregnancy five or more years to 5% of those who did so 2–3 years preceding the survey. The proportion that used non-medical methods remained unchanged at 5% among those who terminated a pregnancy within 3 years preceding the survey. The proportion of women reporting the use of OCP or other, unnamed pills ranged between 10 and 16% over time. A very small proportion (1%-5%) of women reported using injections for induced abortion over time.

Results for the multinomial logistic regression analysis examining factors associated with the abortion method used are presented in Table 2. Compared with older women aged 40–44 years, younger women aged 16–39 years were significantly less likely to use a surgical procedure to induce abortion compared with MA. Compared with Pentecostal Christians, women with no religious affiliation or those who professed traditional religion were significantly less likely to use surgical procedure to terminate pregnancy compared with MA (RRR 0.03; 95% CI 0.1–0.9). Results also show that regardless of the age, women who terminated a pregnancy within the three years preceding the survey had a 60% lower chance of using surgical procedures if they did not use MA compared to those who terminated a pregnancy more than three years before the survey (RRR 0.4; 95% CI 0.3–0.5).

**Table 2:** Relative risk ratios from multinomial logistic regression analysis examining variations in type of abortion methods used by women

Variable	Surgical			Non-medical		
	RRR	95%CI		RRR	95%CI	
*Age*						
16–19	0.1***	0.0	0.4	1.8	0.3	10.6
20–24	0.1***	0.1	0.3	0.5	0.2	1.4
25–29	0.1***	0.1	0.3	0.2***	0.1	0.6
30–34	0.2***	0.1	0.4	0.4**	0.1	0.9
35–39	0.3***	0.2	0.6	0.3**	0.1	0.8
40–44	(ref)					
*Woman’s marital status*						
Currently in union	(ref)					
Never in union	1.0	0.7	1.5	0.9	0.4	1.6
Formerly in union	1.7*	0.9	3.0	2.0	0.9	4.5
*Level of education*						
No formal education	(ref)					
Primary	1.3	0.8	2.2	0.7	0.3	1.4
Middle/junior high school	1.4	0.9	2.3	0.4**	0.2	0.9
Secondary	1.4	0.8	2.4	0.4*	0.2	1.0
Higher	1.7	0.8	3.6	0.7	0.2	3.0
*Religion*						
Pentecostal	(ref)					
Catholic	1.3	0.5	3.7	0.7	0.1	6.8
Anglican/Methodist/Presbyterian	1.0	0.6	1.6	0.7	0.3	2.0
Other Christians	1.1	0.8	1.7	1.6	0.8	3.0
Moslem	0.6	0.3	1.3	0.2	0.0	1.5
Others/No religion	0.3**	0.1	0.9	1.7	0.5	5.4
*Ethnicity*						
Ga/Dangme	(ref)					
Akan	1.1	0.8	1.6	1.0	0.5	1.9
Ewe	0.9	0.6	1.4	1.4	0.7	2.8
Other Ghanaian	1.3	0.7	2.4	0.9	0.2	3.4
Non Ghanaian	1.3	0.2	7.1	6.9*	0.8	62.2
*Wealth Index*						
Poorest	(ref)					
Poorer	1.0	0.6	1.8	0.8	0.4	1.8
Middle	0.9	0.5	1.6	0.4**	0.2	1.0
Richer	0.9	0.5	1.5	0.4*	0.2	1.1
Richest	1.7	0.6	4.7	0.3	0.0	3.0
*Knowledge of abortion law*						
Incorrect Knowledge	(ref)					
Correct knowledge	1.0	0.7	1.5	0.7	0.3	1.6
*Year since last induced abortion*						
< = 3	0.4***	0.3	0.5	0.3***	0.2	0.6
> 3	(ref)					
*Number of observations* = *1018*						
*LR chi2(52)* = *228.2*						
*Prob* > *chi2* = *0.00*						
*Pseudo R2* = *0.1*						

Medication abortion is used as the base category for the dependent variable

*CI *confidence interval;* ref *reference category

***p < 0.01; **p < 0.05; *p < 0.10.

The results further show that women who terminated a pregnancy within the past three years preceding the survey were significantly less likely to use non-medical methods if they did not use MA compared to those who terminated a pregnancy more than three years before the survey (RRR 0.3; 95% CI 0.2–0.6; Table 2). Compared with women from the poorest households, those from households with average wealth had a 60% lower chance of using non-medical methods compared with MA to terminate pregnancy (RRR 0.4; 95% CI: 0.2–1.0). Women with middle or junior high school education were significantly less likely to use non-medical methods if they did not use MA compared to those with no formal education (RRR 0.4; 95% CI 0.2–0.9). The results further show that younger women aged 25–39 years were significantly less likely to use non-medical methods if they did not use MA compared to women aged 40–44 years. Knowledge of abortion law, ethnicity and marital status of women were not significantly associated with the type of method women used to terminate pregnancy.

### Sources of abortion services

**Fig. 3:**
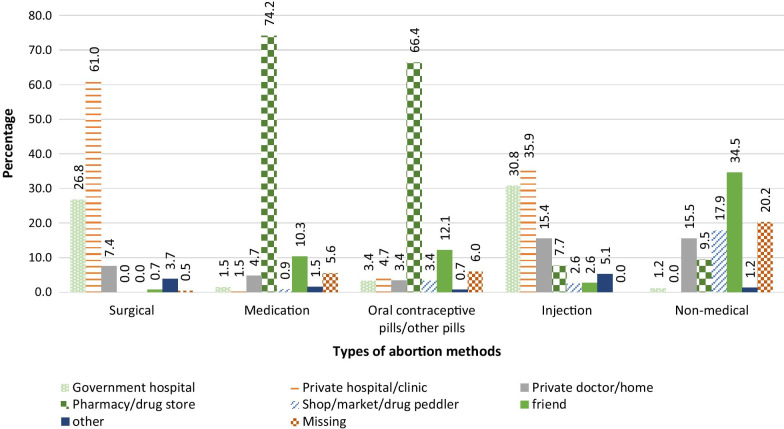
Sources of abortion services/methods

Figure 3 presents the sources of abortion services for women in the study. Majority (61%) of women who used surgical procedures for induced abortion obtained services from private hospitals/clinics, followed by government hospitals (27%). Unlike surgical procedures, majority (74%) of women who used MA obtained services from pharmacies/drug stores. Ten percent of women who used MA obtained the pills from friends. The majority (66%) of women who used OCPs and other unknown pills obtained services from pharmacies/drug stores. Women who terminated a pregnancy using non-medical methods largely obtained services from friends (35%), open markets/drug peddlers/shops (18%), private doctors/home (16%) and pharmacies/drug stores (10%). Private hospitals/clinics (36%) and government health facilities (35%) were the major sources of injections used for pregnancy termination.

## Discussion

The study found that about half of women in urban poor settings of Accra, Ghana who had induced abortion within the past one year used MA. Results show that younger women mostly use MA and do not use surgical procedures as older women. Similarly, younger women in their 20 s and 30 s were significantly less likely to use non-medical methods compared to older women. These findings are consistent with those of a study which found that most university students in Ghana who had an induced abortion used misoprostol tablets [32]. Findings from the 2017 Ghana Maternal Health Survey further showed that over 40% of young women below aged 20 who had an abortion used pharmaceutical tablets containing either misoprostol alone or misoprostol and mifepristone, compared with 27% of women aged 35–49 [19]. Our findings show that women who terminated a pregnancy within the past three years preceding the survey were more likely to use MA than surgical or non-medical procedures compared to those who terminated a pregnancy earlier. The findings suggest women’s changing preferences with increasing access to MA over time. This suggests that safe abortion programs in low-income urban populations in Ghana need to focus on strategies of making registered MA pills widely available, especially for the youth.

We further found a rising trajectory in the use of MA over the past 5 years with a parallel decline in the use of surgical methods while the use of non-medical methods remained low and largely unchanged over time. This is consistent with findings from a study in high-income countries that show increased use of MA over time although the context of our study is different [33]. Our findings suggest that the shift to use MA for pregnancy termination is driven more by a decline in the use of surgical than non-medical methods. In poor urban contexts where there are many pharmacies that serve as the first contact points for health care needs of residents, access to MA may be limited more by stigma associated with abortion and high cost of accessing abortion services than lack of awareness. The findings suggest a need for a review of the abortion laws in Ghana to consider widening the provision of MA by other trained health providers, including pharmacists. Moreover, the provision of MA requires minimal support from trained health providers which could allow the implementation of programs to reduce cost of accessing abortion services and stigma associated with abortion.

Our findings show some variations in the use of non-medical methods by household wealth status, with those from households with average wealth being more likely to use the methods than those from the poorest households. This is, however, contrary to expectations given that those from better-off households may afford safe abortion procedures such as MA or surgical methods compared to those from poorest households. Fear of stigma could be influencing the use of non-medical methods among women who can afford medical abortion services. Our findings further show that women who had no religion or those who adhered to traditional religion were significantly more likely to use medication to terminate a pregnancy compared to those who were Pentecostal Christians. In our data, a higher proportion of women who had no religion or those who professed traditional religion were married compared to Pentecostal Christians. Married women may prefer terminating unintended pregnancies using medication to maintain family cohesion and avoid stigma from society. The results further show that women with middle or junior high school education were more likely to use MA compared to those with no formal education. This was expected as education equips women with information and knowledge, especially through sex education in the school. They are more likely to know of abortion methods and, therefore, able to make an informed choice of a safe abortion method. These findings imply that access to safe abortion services could be improved among poor urban populations through programs that aim at promoting women’s education.

Our findings show that not all surgical procedures are performed in health facilities with the resources and capacity to do so. Although most women who used surgical procedures obtained services from private hospitals/clinics and government health facilities, which are largely licensed service providers, about one-tenth of the surgical procedures were performed at home by private doctors, friends and drug peddlers (unlicensed dealers in drugs which are often illegal). These sources may not meet the minimal medical standards as required by WHO [3], and could endanger the health of women. Fear of stigma and cost of service from qualified providers are possible reasons why women may opt for a surgical procedure in an unsafe environment. According to Aniteye, O’Brien and Mayhew [11], negative attitudes of nurses and midwives stigmatize women seeking abortion services in health facilities. The findings suggest a need for programs that give women opportunities to access safe abortion services in environments where they feel safe.

Our findings further show that MA is accessed mostly through pharmacies and chemists. The regulatory framework in Ghana for the provision of MA does not permit pharmacies to provide the services. In such an environment, it is likely that the high proportion of participants obtaining MA from pharmacies are being provided with products that are either counterfeit or unlicensed. However, pharmacy distribution could be a potential medium for expanding safe abortion services in Ghana. Findings from other developing countries such as Nepal showed that safe MA can be provided if pharmacy workers are trained [17]. Pharmacies are often the first contact points for induced abortion and other family planning services and thus contribute to the provision of essential health services [17, 33, 34]. These findings further support the need for a review of the abortion laws to enable access to safe MA through other health providers.

This study is not without limitations. Since abortion is stigmatized in the sub-Saharan Africa region, there could be underreporting or misreporting of abortion experience. Our survey team was well-trained to develop rapport with the respondents before asking sensitive questions on abortion. Most of our findings are consistent with existing literature in the study region. These are indications that any biases associated with reporting abortion experience in our study may not be different from those of existing studies. It could be argued that women who use MA identify their pregnancies earlier than those who use surgical methods. Since MA is recommended for pregnancies under 10 weeks [35], those who discover that they are pregnant later than 10 weeks or delay their decision to terminate the pregnancy may use surgical abortion. However, our findings suggested that this was not the case as there was no major difference in the proportion using MA by gestational age of the pregnancy. There could also be a tendency for participants to report methods and sources that are licensed or approved by the government. The level of use of non-medical methods in our study may therefore under-estimate actual use. Further, since the study was conducted in Accra, the findings may not be representative of all settings in Ghana. It could also be that women used multiple methods, and we did not specify if they were reporting on their first or last method utilized to have an abortion. We made assumptions that the method chosen was the final method used to terminate the pregnancy. It is possible that some of the women who reported using surgical abortion methods had first used medication, which could result in under-reporting of use of medication to terminate pregnancies. Inferences about causality cannot also be made about our findings since the data are cross-sectional.

## Conclusion

In conclusion, the use of MA pills to terminate pregnancies in poor urban communities in Ghana has increased in recent years and is preferred more by younger women. Medication abortion services are mostly accessed from pharmacies and drug stores in the country. Our findings suggest that the national guidelines for abortion should be updated to include pharmacists, chemists and other trained health providers to support the provision of safe MA services. Programs such as mHealth or telemedicine could also help improve access to safe abortion services among urban-poor women.

## Supplementary Information

**Additional file 1**. Survey guide used for data collection.

## Data Availability

The data used in this paper are available from the corresponding author upon reasonable request.

## Abbreviations

D&C: Dilation and Curettage
D&E: Dilation and Evacuation
EA: Enumeration Area
EVA: Electric Vacuum Aspiration
GMHS: Ghana Maternal Health Survey
GHS: Ghana Health Service
GSS: Ghana Statistical Service
HIPs: High-Impact Practices in Family Planning
IUD: Intrauterine Device
MA: Medication Abortion
MVA: Manual Vacuum Aspiration
OCP: Oral Contraceptive pills
SDG: Sustainable Development Goal
WIE: Willows Impact Evaluation
WHO: World Health Organisation
