# Supplementary material for: Methods women use for induced abortion and sources of services: insights from poor urban settlements of Accra, Ghana
Source: BMC Womens Health. 2021 Aug 16;21:300. doi: 10.1186/s12905-021-01444-9 (PMC8365972; doi:10.1186/s12905-021-01444-9)
Supplement: Supplementary file 1 — Additional file 1. Survey guide used for data collection. [file 12905_2021_1444_MOESM1_ESM.pdf]

WILLOWS EVALUATION STUDY - GHANA
WOMEN'S BASELINE SURVEY, VERSION 2.0 May 2018

GHANA
Principal Investigator: Professor Ayaga Bawah, Regional Institute for Population Studies, University of Ghana

CONSENT

INSTRUCTIONS: TAKE OUT A PAPER COPY OF THE BASELINE WOMEN CONSENT FORM. READ THE CONSENT FORM ALOUD, OR ASK THE WOMAN TO READ THROUGH IT. AT THE END OF THE CONSENT FORM, WHEN IT IS TIME TO COLLECT THE RESPONDENT'S SIGNATURE, ASK THE FOLLOWING QUESTION:

CO1	Do you consent to participate in this study?	YES . . . . 1<br>NO 2	 →**END**

CONSENT FORM SIGNATURES

____
RESPONDENT SIGNATURE OR THUMBPRINT

				2	0	1	
DAY		MONTH		YEAR			

SIGNATURE OF RESPONDENT INDICATES INFORMED CONSENT WAS PROVIDED.

____
ENUMERATOR SIGNATURE

				2	0	1	
DAY		MONTH		YEAR			

SIGNATURE OF ENUMERATOR CONFIRMS THAT INFORMED CONSENT WAS PROVIDED.

RESPONDENT IDENTIFICATION

DISTRICT/METRO/SUBMETRO/MUNICIPALITY [ ][ ]

COMMUNITY . . . . [ ][ ]

CLUSTER NUMBER . . . . [ ][ ][ ]

HOUSEHOLD ID NUMBER . . . . [ ][ ][ ]

HOUSEHOLD LOCATION /DIRECTIONS
____
____
____
____

DESCRIPTION OF HOUSE/ LANDMARKS
____
____

GPS COORDINATES ______________________Latitude ______________________Longitude

RESPONDENT ID NUMBER [ ][ ][ ][ ][ ][ ][ ][ ][ ][ ]

RESPONDENT CONTACT INFORMATION

FULL NAME (INCLUDING COMMON NAME) ______________________

PRIMARY PHONE NO. OF WOMAN ______________________

ALTERNATE PHONE NO. OF WOMAN ______________________

E-MAIL OF WOMAN ______________________

FULL NAME (INCLUDING COMMON NAME) OF FRIEND/RELATIVE ______________________

PHONE NO. OF FRIEND/RELATIVE ______________________

LOCATION/DIRECTIONS TO FRIEND/RELATIVE ______________________

____

____

INTERVIEWER CONTACTS/VISITS

	1	2	3	FINAL CONTACT
DATE	______	______	______	DAY ☐☐<br>MONTH ☐☐<br>YEAR ☐☐☐☐
INTERVIEWER'S NAME	______	______	______	INT. NO. ☐☐☐
RESULT*	______	______	______	RESULT* ☐
NEXT CONTACT: DATE<br>TIME	______<br>______	______<br>______		TOTAL NUMBER OF CONTACTS ☐

*RESULT CODES:
1 COMPLETED
2 NOT AVAILABLE
3 RESCHEDULED
4 REFUSED
5 PARTLY COMPLETED
6 INCAPACITATED
7 OTHER ______________ (SPECIFY)
8 CANNOT LOCATE
9 DECEASED
NOT ELIGIBLE
11 MOVED/MIGRATED
12 BOARDING SCHOOL
13 DOES NOT EXIST

LANGUAGE OF QUESTIONNAIRE** ☐☐ LANGUAGE OF INTERVIEW** ☐☐ NATIVE LANGUAGE OF RESPONDENT** ☐☐

LANGUAGE OF QUESTIONNAIRE** ______________________

**LANGUAGE CODES:
01 ENGLISH 02 GA 03 ASANTI/TWI 04 EWE
05 OTHER:______________________

PLEASE ENTER THE LOCATION OF INTERVIEW
HOME 1
OTHER 2

INTRODUCTION

In this survey I will ask you questions about your health and well-being. The questions in this survey usually take less 60 minutes. All of the answers you give will be confidential and will not be shared with anyone other than members of our research team that will analyze the information collected from all women together. You don't have to be in the survey, but we hope you will agree to answer the questions since your views are important. If I ask you any question you don't want to answer, just let me know and I will go on to the next question or you can stop the interview at any time.

NO.	QUESTIONS AND FILTERS	CODING CATEGORIES	SKIP
001	RECORD THE DATE OF THE INTERVIEW.	DAY . . . .<br>MONTH . . . .<br>YEAR	
002	RECORD THE TIME INTERVIEW BEGINS.	HOURS . . . .<br>MINUTES	

1. HOUSEHOLD ROSTER

						IF AGE 15 OR OLDER	IF AGE 5 YEARS OR OLDER		IF AGE 5- 30YEARS		IF AGE 0-4 YEARS
LINE NO.	USUAL RESIDENTS NAMES	RELATIONSHIP TO WOMAN RESPONDENT	SEX	YEAR OF BIRTH	AGE	MARITAL STATUS	EVER ATTENDED SCHOOL		CURRENT/RECENT SCHOOL ATTENDANCE		BIRTH REGIS-TRATION
101	102	103	104	105	106	107	108	109	110	111	112
	Please give me the names of the persons who usually live in your household, starting with yourself.<br><br>AFTER LISTING THE NAMES AND RECORDING THE RELATIONSHIP AND SEX FOR EACH PERSON, ASK QUESTION **113A** AT THE END OF THE LISTING SECTION TO BE SURE THAT THE LISTING IS COMPLETE.<br><br>THEN ASK APPROPRIATE QUESTIONS 105-112 FOR EACH PERSON.	What is the relationship of (NAME) to you?<br><br>SEE CODES BELOW.<br><br>PROMPT:" What do you call them? She/he is my…"	Is (NAME) male or female?	In what year was (NAME) born?	How old was (NAME) on his/her last birthday?	What is (NAME)'s current marital status?<br><br>1 = MARRIED OR LIVING TOGETHER AS IF MARRIED<br>2 = DIVORCED/ SEPARATED<br>3 = WIDOWED<br>4 = NEVER-MARRIED OR NEVER LIVED TOGETHER<br>9 = REFUSED	Has (NAME) ever attended school?	What is the highest level of school (NAME) has attended?<br><br>SEE CODES BELOW.<br><br>What is the highest grade (NAME) completed?<br><br>SEE CODES BELOW.	Change to Is (NAME) attending school during the 2017-2018 year?	During this school year, what level and grade is (NAME) attending?<br><br>SEE CODES BELOW.	Does (NAME) have a birth certificate?<br><br>IF NO, PROBE:<br>Has (NAME)'s birth ever been registered with the civil authority?<br><br>1 = HAS CERTIFICATE OBSERVED<br>2 = HAS CERTIFICATE NOT OBSERVED<br>3 = REGISTERED<br>4 = NEITHER<br>8 = DON'T KNOW
			M F	YEAR	IN YEARS		Y N	LEVEL GRADE	Y N	LEVEL GRADE	
01			1 2				1 ↓ NEXT LINE	↓	1 2 ↓ NEXT LINE	↓	
02			1 2				1 ↓ NEXT LINE	↓	1 2 ↓ NEXT LINE	↓	
03			1 2				1 ↓ NEXT LINE	↓	1 2 ↓ NEXT LINE	↓	
04			1 2				1 ↓ NEXT LINE	↓	1 2 ↓ NEXT LINE	↓	
05			1 2				1 ↓ NEXT LINE	↓	1 2 ↓ NEXT LINE	↓	
06			1 2				1 ↓ NEXT LINE	↓	1 2 ↓ NEXT LINE	↓	
07			1 2				1 ↓ NEXT LINE	↓	1 2 ↓ NEXT LINE	↓	
08			1 2				1 ↓ NEXT LINE	↓	1 2 ↓ NEXT LINE	↓	
09			1 2				1 ↓ NEXT LINE	↓	1 2 ↓ NEXT LINE	↓	
10			1 2				1 ↓ NEXT LINE	↓	1 2 ↓ NEXT LINE	↓	

CODES FOR Q. 103: RELATIONSHIP TO WOMAN RESPONDENT

01 = SELF	08 = BROTHER OR SISTER	16=COUSIN
02 = HUSBAND	09 = OTHER RELATIVE	88=DON'T KNOW
03 = SON OR DAUGHTER	10 = ADOPTED/FOSTER/ STEPCHILD	99=REFUSE
04 = SON-IN-LAW OR DAUGHTER-IN-LAW	11 = NOT RELATED	
05 = GRANDCHILD	12=GRANDPARENT	
06 = PARENT	13=BROTHER IN LAW/SISTER IN LAW	
07 = PARENT-IN-LAW	14=NIECE/NEPHEW	
	15= AUNT/UNCLE	

CODES FOR Qs. 109 AND 111: EDUCATION

LEVEL
0= NONE
1 = PRIMARY
2 = MIDDLE/JHS/JSS
3 = SECONDARY
4 = HIGHER
8 = DON'T KNOW
9 = REFUSED
10 = OTHER

GRADE
90 = No level completed

80 = Pre-school	06 = P6	13 = JSS1/JHS3	21 = S3	28 = Teacher Training
00 = Kindergarten	07 = M1	14 = SS1/SHS1	22 = S4	29 = Nursing
01 = P1	08 = M2	15 = SS2/SHS2	23 = S5	30 = Polytechnic
02 = P2	09 = M3	16 = SS3/SHS3		31 = University
03 = P3	10 = M4	18 = SHS4	25 = L6	32 = Other
04 = P4	11 = JSS1/JHS1	19 = S1	26 = U6	88 = Don't know
05 = P5	12 = JSS1/JHS2	20 = S2	27 = Voc/Tech/Agric/Computer	99 = Refused

			M F	YEAR	IN YEARS		Y N	LEVEL	GRADE	Y N	LEVEL	GRADE	
11			1 2				1 NEXT LINE			1 2 NEXT LINE			
12			1 2				1 NEXT LINE			1 2 NEXT LINE			
13			1 2				1 NEXT LINE			1 2 NEXT LINE			
14			1 2				1 NEXT LINE			1 2 NEXT LINE			
15			1 2				1 NEXT LINE			1 2 NEXT LINE			
16			1 2				1 NEXT LINE			1 2 NEXT LINE			
17			1 2				1 NEXT LINE			1 2 NEXT LINE			
18			1 2				1 NEXT LINE			1 2 NEXT LINE			
19			1 2				1 NEXT LINE			1 2 NEXT LINE			
20			1 2				1 NEXT LINE			1 2 NEXT LINE			

TICK HERE IF CONTINUATION SHEET USED ☐

113A) Just to make sure that I have a complete listing: are there any other persons such as small children or infants that we have not listed?

YES ☐ → [illegible] TO TABLE NO ☐

2. HOUSEHOLD CHARACTERISTICS

NO.	QUESTIONS AND FILTERS	CODING CATEGORIES	SKIP
201	Now I would like to ask you questions about your household.<br>What is the main source of drinking water for members of your household?	PIPED WATER 1<br>WELL OR BOREHOLE . . . . 2<br>TANKER TRUCK/CART . . . . 3<br>RAIN/SURFACE WATER (RIVER/DAM) 4<br>BOTTLED/SACHET WATER 5<br>OTHER ______________________ 96<br>(SPECIFY)<br>REFUSED 99	
202	What kind of toilet facility do members of your household usually use?	FLUSH OR POUR FLUSH TOILET . . . . 1<br>PIT LATRINE . . . . 2<br>BUCKET TOILET . . . . 3<br>PUBLIC TOILET (KVIP) . . . . 4<br>NO FACILITY/BUSH/FIELD/S . . . . 5<br>OTHER ______________________ 96<br>(SPECIFY)<br>REFUSED 99	4, 5 → 205
203	Do you share this toilet facility with other households?	YES . . . . 1<br>NO . . . . 2<br>REFUSED 99	2 → 205
204	How many households use this toilet facility (including your own)?	NO. OF HOUSEHOLDS . . . . [ ][ ]<br>DON'T KNOW . . . . 88<br>REFUSED 99	
205	Does your household have:<br><br>Electricity?<br>A wall clock?<br>A working television?<br>A cellular/mobile phone?<br>HOW MANY CELL/MOBILE PHONES?<br>A refrigerator?<br>A working video deck/DVD/VCR?<br>A Cabinet/cupboard (including a wardrobe or wall divider)?<br>An MP3/MP4 player/iPod?<br>Working electric or box iron?<br>A microwave<br>A ricecooker<br>A decoder (DSTV, MultiTV, Startime, GoTV, KweseTV)<br>A computer or laptop?<br>A wrist watch?	YES NO DK RF<br>1 ELECTRICITY . . . . 1 2 88 99<br>2 WALL CLOCK . . . . 1 2 88 99<br>3 TELEVISION . . . . 1 2 88 99<br>4 CELL PHONE . . . . 1 2 88 99<br>5 NUMBER OF CELL PHONES [ ][ ] 88 99<br>6 REFRIGERATOR . . . . 1 2 88 99<br>7 DVD VCR . . . . 1 2 88 99<br>8 CABINET . . . . 1 2 88 99<br>9 MP3 . . . . 1 2 88 99<br>10 IRON . . . . 1 2 88 99<br>11 MICROWAVE ………………….. 1 2 88 99<br>12 RICECOOKER………………….. 1 2 88 99<br>13 DECODER……………………… 1 2 88 99<br>14 COMPUTER 1 2 88 99<br>15 WATCH 1 2 88 99	
206	What type of fuel does your household mainly use for cooking?	ELECTRICITY . . . . 1<br>WOOD, CROP RESIDUE, SAWDUST, OR ANIMAL WASTE 2<br>KEROSENE OR CHARCOAL 3<br>LPG/GAS 4<br>NO FOOD COOKED IN HOUSEHOLD 95<br>OTHER ______________________ 96<br>(SPECIFY)<br>REFUSED 99	95 → 209
207	Is the cooking usually done in the house, in a separate building, or outdoors?	IN THE HOUSE . . . . 1<br>IN A SEPARATE BUILDING . . . . 2<br>OUTDOORS . . . . 3<br>OTHER ______________________ 6<br>(SPECIFY)<br>REFUSED 99	2, 3, 6 → 209
208	Do you have a separate room which is used as a kitchen?	YES . . . . 1<br>NO . . . . 2<br>REFUSED 99	

209	MAIN MATERIAL OF THE FLOOR.<br>RECORD OBSERVATION, IF POSSIBLE<br>OR ENQUIRE WHEN OBSERVATION IS NOT POSSIBLE	NATURAL FLOOR (EARTH OR DUNG) 1<br>RUDIMENTARY FLOOR (WOOD OR BAMOC 2<br>FINISHED FLOOR (CEMENT/CERAMIC/CAR 3<br><br>OTHER ______________ 96<br>(SPECIFY)<br>NOT OBSERVED / NOT REPORTED 99	
210	MAIN MATERIAL OF THE ROOF.<br>RECORD OBSERVATION, IF POSSIBLE<br>OR ENQUIRE WHEN OBSERVATION IS NOT POSSIBLE	NATURAL ROOFING (NONE OR THATCH) 1<br>RUDIMENTARY ROOFING (CARDBOARD/W 2<br>FINISHED ROOFING (METAL/TILE/CERAMIC 3<br><br>OTHER ______________ 96<br>(SPECIFY)<br>NOT OBSERVED / NOT REPORTED 99	
211	MAIN MATERIAL OF THE EXTERIOR WALLS.<br>RECORD OBSERVATION, IF POSSIBLE<br>OR ENQUIRE WHEN OBSERVATION IS NOT POSSIBLE	NATURAL WALLS (CANE/PALM/TRUNKS/NO 1<br>RUDIMENTARY WALLS (MUD/PLASTER/WO 2<br>FINISHED WALLS (CEMENT/STONE/BRICK) 3<br><br>OTHER ______________ 96<br>(SPECIFY)<br>NOT OBSERVED / NOT REPORTED 99	
212	How many rooms in this household are used for sleeping?	ROOMS . . . . [ ][ ]<br>NOT OBSERVED / NOT REPORTED 99	
213	Does any member of this household own:<br><br>A bicycle?<br>A motorcycle or motor scooter?<br>A motorking?<br>An animal-drawn cart (oxcart)?<br>A car or truck?<br>A boat with a motor?	YES NO DK RF<br><br>2 BICYCLE . . . . 1 2 88 99<br>3 MOTORCYCLE/SCOOTER 1 2 88 99<br>4 MOTORKING 1 2 88 99<br>5 ANIMAL-DRAWN CART . . . . . 1 2 88 99<br>6 CAR/TRUCK . . . . 1 2 88 99<br>7 BOAT WITH MOTOR 1 2 88 99	
214	Does any member of this household own any agricultural land?	YES . . . . 1<br>NO . . . . 2<br>DON'T KNOW . . . . 88<br>REFUSED 99	NO / DON'T KNOW / REFUSED → 216
215	How much agricultural land do members of this household own?<br><br>RECORD IN UNITS RESPONDENT USES	HECTARES . . . . 1 [ ][ ].[ ]<br><br>ACRES . . . . 2 [ ][ ].[ ]<br><br>PLOTS . . . . 3 [ ][ ].[ ]<br><br>DON'T KNOW . . . . 998<br>REFUSED 999	
216	Does any member of this household have a bank account?	YES . . . . 1<br>NO . . . . 2<br>DON'T KNOW . . . . 88<br>REFUSED 99	
217	Does someone in your household own your home?	YES . . . . 1<br>NO . . . . 2<br>REFUSED 99	

3. RESPONDENT'S BACKGROUND

NO.	QUESTIONS AND FILTERS	CODING CATEGORIES	SKIP
303	In which region or country were you born?	Western . . . 1<br>Central . . . 2<br>Greater Accra . . . 3<br>Volta . . . 4<br>Eastern . . . 5<br>Ashanti . . . 6<br>Brong Ahafo . . . 7<br>Northern . . . 8<br>Upper East . . . 9<br>Upper West . . . 10<br>Nigeria . . . 11<br>Liberia . . . 12<br>Sierra Leone . . . 13<br>Gambia . . . 14<br>Togo . . . 15<br>Burkina Faso . . . 16<br>Cote d'Ivoire . . . 17<br>Other ECOWAS state . . . 18<br>Africa other t . . . 19<br>Europe . . . 20<br>Americas . . . 21<br>Asia . . . 22<br>Oceania . . . 23<br>Other (not listed): ______ 96<br>Refused . . . 99	
304	For how long have you resided in this neighborhood/area?	MONTHS . . . [ ][ ]<br>YEARS . . . [ ][ ]<br>DON'T KNOW . . . 888<br>REFUSED . . . 999	
305	What is your religion?	CATHOLIC . . . 1<br>ANGLICAN . . . 2<br>METHODIST . . . 3<br>PRESBYTERIAN . . . 4<br>PENTECOSTAL/CHARISMAT . . . 5<br>OTHER CHRISTIAN . . . 6<br>MOSLEM . . . 7<br>TRADITIONAL/SPIRITUALIST . . . 8<br>NO RELIGION . . . 9<br>OTHER______ 96<br>(SPECIFY)<br>REFUSED . . . 99	
G_3_1	How often do you attend religious services?	NEVER . . . 9<br>MORE THAN ONCE PER WEEK . . . 1<br>ONCE PER WEEK . . . 2<br>NEARLY EVERY WEEK . . . 3<br>2-3 TIMES PER MONT . . . 4<br>ABOUT ONCE PER MONTH . . . 5<br>SEVERAL TIMES PER YEAR . . . 6<br>ONCE OR TWICE A YEAR . . . 7<br>LESS THAN ONCE A YEAR . . . 8<br>REFUSED . . . 99	
306	What is your tribe or ethnic group?	AKAN . . . 1<br>GA/DANGME . . . 2<br>EWE . . . 3<br>GUAN . . . 4<br>MOLE-DAGBANI . . . 5<br>GRUSI . . . 6<br>GURMA . . . 7<br>MANDE . . . 8<br>OTHER______ 96<br>(SPECIFY)<br>REFUSED . . . 99	

4. REPRODUCTION

NO.	QUESTIONS AND FILTERS	CODING CATEGORIES		SKIP
401	Now I will read you a list of statements, and I would like you to tell me how many of the statements are true for you. You should not tell me which statements are true, only how many. By giving only the number of statements that are true for you, this will preserve your privacy as I will not know which statements are true for you. After I read the list, please tell me how many of these statements are true for you. First, we will complete an example:<br><br>1. I have three children.<br>2. I am 22 years old.<br>3. I have received antenatal care during a pregnancy.<br>4. I have had a tetanus toxoid vaccination.<br><br>*How many* of these statements are true for you?	NUMBER OF STATEMENTS<br>REFUSED	 99	
402	Do you have any questions about how to answer these questions?	YES . . . .<br>NO	1<br>2	1 → ANSWER QUESTIONS
403	Now we will complete the first real list of statements about your health. I will read you the list of statements, and I would like you to tell me how many of these statements are true for you.<br><br>1. I have heard of an illness called tuberculosis (TB)<br>2. I have been told I have hypertension or high blood pressure<br>3.I have ever smoked cigarettes<br>4. I had malaria as a child<br>5. I have had an induced abortion (ended a pregnancy on purpose)<br><br>*How many* of these statements are true for you?	NUMBER OF STATEMENTS<br>REFUSED	 99	
404	Here is the next list of statements about your health. Please tell me how many of these statements are true for you.<br><br>1. I have had a cold in the last year<br>2. I have heard of an illness called diabetes (high blood sugar)<br>3. I know someone who has told me they are HIV-positive<br>4. I have been diagnosed with cancer<br>5. I have had an induced abortion (ended a pregnancy on purpose)<br><br>*How many* of these statements are true for you?	NUMBER OF STATEMENTS<br>REFUSED	 99	
G_4_1	Now I would like to ask some questions about marriage and sexual activity in order to gain a better understanding of some important life issues.<br><br>**CHECK 107:**<br>Just to make sure that I have this right: your current marital status is _________________________. Is that correct?<br>*If NOT correct, please change the roster information and return here.*<br><br>EVER MARRIED ↓<br>*(Options 1,2 or 3)*	NEVER MARRIED OR NEVER LIVED TOGETHER, OR REFUSED<br>*Option 4 or 9*		406
405	How old were you when you first got married or started living with your partner as if married?	AGE IN COMPLETED YEARS<br>DON'T KNOW . . . .<br>REFUSED	 88<br>99	

4. REPRODUCTION

NO.	QUESTIONS AND FILTERS	CODING CATEGORIES	SKIP
406	How old were you when you had sexual intercourse for the very first time?	NEVER HAD SEXUAL INTERCOUF . . . . 0<br>AGE IN YEARS . . . . [ ][ ]<br>. . . . .<br>REFUSED 99	→ 501<br><br><br>→ 408
407	When was the last time you had sexual intercourse? (RECORD IN UNIT RESPONDENT USES)<br>*If less than 12 months ago, answer must be recorded in months, weeks, or days. Enter 0 days for today.*	[ ][ ] Days ago<br>[ ][ ] Weeks ago<br>[ ][ ] Months ago<br>[ ][ ] Years ago<br>DON'T KI . . . . 88<br>REFUSE 99	
408	Now I would like to ask about all the live births you have had during your life. Have you ever given birth?	YES . . . . 1<br>NO . . . . 2<br>REFUSED 99	→ 420
409	How old were you when you gave birth for the first time?	AGE IN YEARS [ ][ ]<br>DON'T KNOW 88<br>REFUSED 99	
410	Do you have any sons or daughters to whom you have given birth who are now living with you?	YES . . . . 1<br>NO . . . . 2<br>REFUSE 99	→ 412
411	How many sons live with you?<br>And how many daughters live with you?<br>IF NONE, RECORD '00'.<br>IF REFUSED, RECORD '99'	SONS AT HOME . . . . [ ][ ]<br>DAUGHTERS AT HOME [ ][ ]	
412	Do you have any sons or daughters to whom you have given birth who are alive but do not live with you?	YES . . . . 1<br>NO . . . . 2<br>REFUSE 99	→ 414
413	How many sons are alive but do not live with you?<br>And how many daughters are alive but do not live with you?<br>IF NONE, RECORD '00'.<br>IF REFUSED, RECORD '99'	SONS ELSEWHERE [ ][ ]<br>DAUGHTERS ELSEWHERE [ ][ ]	
414	Have you ever given birth to a boy or girl who was born alive but later died?<br>IF NO, PROBE: Any baby who cried or showed signs of life but did not survive?	YES . . . . 1<br>NO . . . . 2<br>REFUSE 99	→ 416
415	How many boys have died?<br>And how many girls have died?<br>IF NONE, RECORD '00'.<br>IF REFUSED, RECORD '99'	BOYS DEAD . . . . [ ][ ]<br>GIRLS DEAD . . . . [ ][ ]	
416	SUM ANSWERS TO 411, 413, AND 415, AND ENTER TOTAL.<br>IF NONE, RECORD '00'.<br>IF REFUSED, RECORD '99'	TOTAL BIRTHS . . . . [ ][ ]	

4. REPRODUCTION

NO.	QUESTIONS AND FILTERS	CODING CATEGORIES	SKIP
417	CHECK 416:<br>Just to make sure that I have this right: you have had in TOTAL _____ births during your life. Is that correct?<br>YES ☐ ↓ NO ☐ →		411
418	CHECK 416:<br>ONE OR MORE BIRTHS ☐ ↓ NO BIRTHS ☐ →		420
419	**C** FOR EACH BIRTH SINCE JULY 2015 **(Column 1)**, ENTER 'B' IN THE MONTH OF BIRTH IN THE CALENDAR. WRITE THE NAME OF THE CHILD TO THE LEFT OF THE 'B' CODE. FOR EACH BIRTH, ASK THE NUMBER OF MONTHS THE PREGNANCY LASTED AND RECORD 'P' IN EACH OF THE PRECEDING MONTHS ACCORDING TO THE DURATION OF PREGNANCY. (NOTE: THE NUMBER OF 'P's MUST BE ONE LESS THAN THE NUMBER OF MONTHS THAT THE PREGNANCY LASTED.)		
420	Now I would like to ask you some questions about pregnancy. Are you pregnant now?	YES . . . . 1<br>NO . . . . 2<br>DON'T KNOW / UNSURE 88<br>REFUSE 99	if 418=1 to 424<br>if 418=0 425
421	How many months pregnant are you?<br>RECORD NUMBER OF COMPLETED MONTHS.<br>**C** ENTER 'P's IN THE CALENDAR, BEGINNING WITH THE MONTH OF INTERVIEW AND FOR THE TOTAL NUMBER OF COMPLETED MONTHS.	MONTHS . . . . . . ☐☐<br>DON'T KNOW . . . . 88<br>REFUSE 99	
422	When you got pregnant, did you want to get pregnant at that time?	YES . . . . 1<br>NO . . . . 2<br>DON'T KNOW . . . . 88<br>REFUSE 99	1 → 425
423	Did you want to have a baby later on or did you not want any more children?	LATER . . . . 1<br>NO MORE/NONE . . . . 2<br>DON'T KNOW . . . . 88<br>REFUSE 99	→ 425
424	Have you given birth within the last six months?	YES . . . . 1<br>NO . . . . 2<br>REFUSE 99	
425	Have you ever had a pregnancy that miscarried, was aborted, or ended in a stillbirth (no signs of life)?	YES . . . . 1<br>NO . . . . 2<br>REFUSE 99	2, 99 → 501
426	When did the last such pregnancy end?<br>(RECORD IN UNIT RESPONDENT USES)	☐☐ Days ago<br>☐☐ Weeks ago<br>☐☐ Months ago<br>☐☐ Years ago<br>DON'T KNOW 88<br>REFUSED 99	
427	How many pregnancies were miscarried, ended by induced abortion, or ended in a stillbirth?	NUMBER OF PREGNANCY LOSSES ☐☐<br>REFUSE 99	
428	How many pregnancies ended in a stillbirth?	NUMBER OF STILLBIRTHS ☐☐<br>NONE . . . . 00<br>DON'T KNOW . . . . 88<br>REFUSE 99	
429	How many pregnancies were miscarried?	NUMBER OF MISCARRIAGES ☐☐<br>NONE . . . . 00<br>DON'T KNOW . . . . 88<br>REFUSE 99	

4. REPRODUCTION			
NO.	QUESTIONS AND FILTERS	CODING CATEGORIES	SKIP
430	How many pregnancies were terminated by induced abortion (intentional termination of pregnancy)?	NUMBER OF ABORTIONS [ ][ ]<br>NONE . . . . 00<br>DON'T KNOW .... 88<br>REFUSE 99	00 → 444
431	When was the last induced abortion?<br><br>(RECORD IN UNIT RESPONDENT USES)	[ ][ ] Days ago<br>[ ][ ] Weeks ago<br>[ ][ ] Months ago<br>[ ][ ] Years ago<br>DON'T KI . . . . 88<br>REFUSE 99	
435	What was done to have the pregnancy terminated?	SURGICALLY (OPERATION) 1<br>INJECTION 2<br>TOOK PILLS/MEDICINES/MEDICATION ABORTION (M 3<br>INSERTED HERBS OR OBJECT IN WOMB, SPECIFY_ 4<br>TOOK HOMEMADE MEDICINE SPECIFY ___________ 5<br>OTHER, SPECIFY 96<br>REFUSE 99	1, 2 → 439<br>3 → 437<br>4, 5, 96, 99 → 439
436	What was the name of the surgical procedure used to end the pregnancy?	VACUUM ASPIRATION (MVA or EVA) 1<br>DILATION AND CURETTAGE (D&C)/<br>SHARP CURETTAGE 2<br>IUD INSERTION 3<br>OC PILLS/ TABLET 4<br>OTHER, SPECIFY 96<br>DON'T KNOW 88<br>REFUSE 99	→ 439
437	What kind of medication did you use to end the pregnancy?	MIFEPRISTONE&MISOPROSTOL<br>(MEDABON/MARIPRIST) 1<br>MISOPROSTOL ALONE (MISOCLEAR/CYTOTEC) 2<br>ORAL CONTRACEPTIVE PILLS (Lydia, Microgynon) 3<br>OTHER PILLS 4<br>N_TABLETS 5<br>OTHER, SPECIFY 96<br>DON'T KNOW 88<br>REFUSE 99	96, 88, 99 → 439
437B	How did you take the pills?	___________________________<br>ENTER "DON'T KNOW or REFUSE" in TEXT I FNEEDED	
438	In total, how many tablets or pills did you take to end the pregnancy?	NUMBER OF PILLS [ ][ ]<br>NOT APPLICABLE NA<br>DON'T KNOW 88<br>REFUSED 99	

4. REPRODUCTION			
NO.	QUESTIONS AND FILTERS	CODING CATEGORIES	SKIP
432	From whom/where did you receive induced abortion services the last time?<br><br>SELECT ALL THAT APPLY	PUBLIC SECTOR<br>GOVT. HOSPITAL/POLYCLINIC . . . . 11<br>GOVT. HEALTH CENTER/CLINIC . . . . 12<br>GOVT. HEALTH POST/CHPS . . . . 13<br>FAMILY PLANNING CLINIC . . . . 14<br>MOBILE CLINIC 15<br>FIELDWORKER/OUTREACH/ PEER EDUCATOR 16<br>OTHER PUBLIC ___________ 17<br>(SPECIFY)<br><br>PRIVATE MEDICAL SECTOR<br>PRIVATE HOSPITAL/CLINIC . . . . 21<br>PRIVATE DOCTOR 22<br>PHARMACY . . . . 23<br>CHEMICAL/DRUG STORE . . . . 24<br>FP/PPAG CLINIC 25<br>MATERNITY HOME 26<br>PROVIDER's HOME 37<br>OTHER PRIVATE MEDICAL ___________ 27<br>(SPECIFY)<br><br>OTHER SOURCE<br>SHOP/MARKET 31<br>COMMUNITY VOLUNTEER . . . . 33<br>FRIEND/RELATIVE/PARTNER 34<br>NO SERVICES OBTAINED . . . . 35<br>ITINERANT DRUG PEDDLER 36<br>OTHER ___________ 96<br>(SPECIFY)<br>REFUSED 99	
433	Where did you learn about this place (primary source of information)?<br><br>Select only one (Probe for primary source)	NEIGHBOR 1<br>FRIEND 2<br>MOTHER 3<br>MOTHER-IN-LAW 4<br>HUSBAND/ PARTNER 5<br>OTHER RELATIVE 6<br>DOCTOR 7<br>NURSE/MIDWIFE WORKING AT HEALTH FACILITY 8<br>COMMUNITY HEALTH WORKER MAKING A HOME VI 9<br>FIELD WORKER MAKING A HOME VISIT 10<br>PHARMACIST 11<br>NO ONE 12<br>OTHER ___________ 96<br>(SPECIFY)<br>DON'T KNOW . . . . 88<br>REFUSE 99	
434	Who influenced you the most when deciding on this place? (Select one)	NEIGHBOR 1<br>FRIEND 2<br>MOTHER 3<br>MOTHER-IN-LAW 4<br>HUSBAND/PARTNER 5<br>OTHER RELATIVE 6<br>DOCTOR 7<br>NURSE/MIDWIFE WORKING AT HEALTH FACILITY 8<br>COMMUNITY HEALTH WORKER MAKING A HOME VI 9<br>FIELD WORKER MAKING A HOME VISIT 10<br>PHARMACIST 11<br>NO ONE 12<br>OTHER ___________ 96<br>(SPECIFY)<br>DON'T KNOW . . . . 88<br>REFUSE 99	
440	After termination, did you experience any health problems? (Select all that apply)	HEAVY BLEEDING 1<br>VOMITING/NAUSEA 2<br>ABDOMINAL PAIN 3<br>FEVER 4<br>FOUL-SMELLING DISCHARGE/INFECTION 5<br>NO HEALTH PROBLEMS 6<br>OTHER, SPECIFY ___________ 96<br>REFUSED 99	6 → Section 5
439	Did you seek care or treatment for any of these problems?	YES 1<br>NO 2<br>REFUSED 99	2, 99 → Section 5

4. REPRODUCTION

NO.	QUESTIONS AND FILTERS	CODING CATEGORIES	SKIP
441	Where did you receive treatment for these problems?<br><br>SELECT ALL THAT APPLY	PUBLIC SECTOR<br>GOVT. HOSPITAL/POLYCLINIC . . . . 11<br>GOVT. HEALTH CENTER/CLINIC . . . . 12<br>GOVT. HEALTH POST/CHPS . . . . 13<br>FAMILY PLANNING CLINIC . . . . 14<br>MOBILE CLINIC 15<br>FIELDWORKER/OUTREACH/ PEER EDUCATOR 16<br>OTHER PUBLIC ____________ 17<br>(SPECIFY)<br><br>PRIVATE MEDICAL SECTOR<br>PRIVATE HOSPITAL/CLINIC . . . . 18<br>PRIVATE DOCTOR 19<br>PHARMACY . . . . 20<br>CHEMICAL/DRUG STORE . . . . 21<br>FP/PPAG CLINIC 22<br>MATERNITY HOME 23<br>OTHER PRIVATE MEDICAL ____________ 24<br>(SPECIFY)<br><br>OTHER SOURCE<br>SHOP/MARKET 25<br>COMMUNITY VOLUNTEER . . . . 26<br>FRIEND/RELATIVE 27<br>OTHER ____________ 96<br>(SPECIFY)<br>DON'T KNOW 88<br>REFUSED 99	
442	Who influenced you the most when deciding on this place?	NEIGHBOR 1<br>FRIEND 2<br>MOTHER 3<br>MOTHER-IN-LAW 4<br>HUSBAND/PARTNER 5<br>OTHER RELATIVE 6<br>DOCTOR 7<br>NURSE/MIDWIFE WORKING AT HEALTH FACILITY 8<br>COMMUNITY HEALTH WORKER MAKING A HOME VI 9<br>FIELD WORKER MAKING A HOME VISIT 10<br>PHARMACIST 11<br>NO ONE 12<br>OTHER ____________ 96<br>(SPECIFY)<br>DON'T KNOW . . . . 88<br>REFUSE 99	
443	Did you receive any family planning counseling during this visit?	YES 1<br>NO 2<br>DON'T KNOW 88<br>REFUSED 99	
444	**C** FOR EACH PREGNANCY THAT DID NOT END IN A LIVE BIRTH IN JULY 2015 **(Col. 1)** OR LATER, ENTER 'T' IN THE CALENDAR IN THE MONTH THAT THE PREGNANCY TERMINATED AND 'P' FOR THE REMAINING NUMBER OF COMPLETED MONTHS OF PREGNANCY.<br><br>IF THERE ARE MORE THAN FOUR PREGNANCIES THAT DID NOT END IN A LIVE BIRTH,		

(1) Year of fieldwork is assumed to be 2018. For fieldwork beginning in 2019 or 2020, the year should be adjusted.

**5. FAMILY PLANNING**			
NO.	QUESTIONS AND FILTERS	CODING CATEGORIES	SKIP
501	Now I would like to talk about family planning - the various ways or methods that a man and woman can use to delay or avoid a pregnancy. Have you heard of:		
1	Female Sterilization. PROBE: Women can have an operation to avoid having any more children.	YES . . . . . 1<br>NO . . . . . 2<br>REFUSED 99	
2	Male Sterilization. PROBE: Men can have an operation to avoid having any more children.	YES . . . . . 1<br>NO . . . . . 2<br>REFUSED 99	
3	IUD. PROBE: Women can have a loop or coil placed inside them by a doctor or a nurse. (wearing loop or wearing rubber)	YES . . . . . 1<br>NO . . . . . 2<br>REFUSED 99	
4	Injectables. PROBE: Women can have an injection by a health provider that stops them from becoming pregnant for one or more months.	YES . . . . . 1<br>NO . . . . . 2<br>REFUSED 99	
5	Implants. PROBE: Women can have one or more small rods placed in their upper arm by a doctor or nurse which can preventy pregnancy for one or more years.	YES . . . . . 1<br>NO . . . . . 2<br>REFUSED 99	
6	Pill. PROBE: Women can take a pill every day to avoid becomine pregnant.	YES . . . . . 1<br>NO . . . . . 2<br>REFUSED 99	
7	Condom. PROBE: Men can put a rubber sheath on their penis before sexual intercourse to prevent pregnancy.	YES . . . . . 1<br>NO . . . . . 2<br>REFUSED 99	
8	Female Condom. PROBE: Women can place a sheath in their vagina before sexual intercourse to prevent pregnancy.	YES . . . . . 1<br>NO . . . . . 2<br>REFUSED 99	
9	Spermicides/Foam tablets. PROBE: Women can place a suppository, jelly or cream in their vagina before sexual intercourse to prevent pregnancy.	YES . . . . . 1<br>NO . . . . . 2<br>REFUSED 99	
10	Lactational Amenorrhea Method (LAM). PROBE: Women can exclusively breastfeed their children until 6 months of age to avoid pregnancy.	YES . . . . . 1<br>NO . . . . . 2<br>REFUSED 99	
11	Rhythm/Calendar Method. PROBE: To avoid pregnancy, women do not have sexual intercourse on the days of the month they think they can get pregnant.	YES . . . . . 1<br>NO . . . . . 2<br>REFUSED 99	
12	Withdrawal. PROBE: Men can be careful and pull out before climax.	YES . . . . . 1<br>NO . . . . . 2<br>REFUSED 99	
13	Emergency Contraception. PROBE: As an emergency measure, within three days after they have unprotected sexual intercourse, women can take special pills to prevent pregnancy.	YES . . . . . 1<br>NO . . . . . 2<br>REFUSED 99	

5. FAMILY PLANNING			
NO.	QUESTIONS AND FILTERS	CODING CATEGORIES	SKIP
14	Have you heard of any other ways or methods that women or men can use to avoid pregnancy?	YES . . . . . 1<br>________ (SPECIFY)<br>________ (SPECIFY)<br>NO . . . . . 2<br>REFUSED 99	
502	IF "NO" TO ALL of 501 SKIP.<br>Where did you learn about any of these family planning methods?<br>SELECT ALL THAT APPLY	NEIGHBOR 1<br>FRIEND 2<br>RELATIVE 3<br>HUSBAND 4<br>DOCTOR 5<br>NURSE/MIDWIFE WORKING AT HEALTH FACILITY 6<br>NURSE/MIDWIFE MAKING A HOME VISIT 7<br>FIELD WORKER MAKING A HOME VISIT 8<br>PHARMACIST 9<br>BOOK 10<br>NEWSPAPER OR MAGAZINE 11<br>TELEVISION 12<br>INTERNET 13<br>SOCIAL MEDIA 14<br>NO ONE 15<br>BILLBOARD 16<br>OTHER ________ 96<br>(SPECIFY)<br>DON'T KNOW . . . . . 88<br>REFUSED 99	
503	According to Ghanaian law, under what condition(s) is it legal to have an induced abortion?<br>CIRCLE ALL THOSE MENTIONED SPONTANEOUSLY BY THE RESPONDENT	On request 1<br>Economic or social reasons 2<br>Foetal impairment 3<br>Rape 4<br>Incest 5<br>Intellectual or cognitive disability of the woman 6<br>Mental Health 7<br>Physical Health 8<br>Under no conditions/NOT LEGAL 9<br>Other ________<br>Don't know 88<br>Refused 99	 <br><br><br><br><br><br><br>SKIP TO 50<br><br>SKIP TO 50<br>SKIP TO 50
504	According to Ghanaian law, up to how many weeks of pregnancy is it permitted to have an induced abortion?	WEEKS . . . . . [ ][ ]<br>DON'T KNOW . . . . . 88<br>REFUSED 99	
505	What methods for induced abortion have you ever heard of?<br>SELECT ALL THAT APPLY.	SURGERY/OPERATION . . . . . 1<br>TABLETS/PILLS/MEDICATION ABORTION (MA) . . 2<br>HOMEMADE CONCOCTION . . . . . 3<br>INSERTING HERBS OR OBJECT IN WOMB . . . . 4<br>MASSAGE . . . . . 5<br>NONE . . . . . 6<br>INJECTION 7<br>OTHER ________ 96<br>(SPECIFY)<br>REFUSED 99	
506	Have you ever sought information about having an induced abortion for an unwanted pregnancy?	YES . . . . . 1<br>NO . . . . . 2<br>DON'T KNOW . . . . . 88<br>REFUSED 99	 NO, DON'T KNOW, REFUSED → 508

5. FAMILY PLANNING

NO.	QUESTIONS AND FILTERS	CODING CATEGORIES	SKIP
507	Where did you get information about induced abortion?<br><br>SELECT ALL THAT APPLY	NEIGHBOR 1<br>FRIEND 2<br>RELATIVE 3<br>HUSBAND 4<br>DOCTOR 5<br>NURSE/MIDWIFE WORKING AT HEALTH FACILITY 6<br>NURSE/MIDWIFE MAKING A HOME VISIT 7<br>FIELD WORKER MAKING A HOME VISIT 8<br>PHARMACIST 9<br>BOOK 10<br>NEWSPAPER OR MAGAZINE 11<br>TELEVISION 12<br>INTERNET 13<br>SOCIAL MEDIA 14<br>NO ONE 15<br>OTHER ______ 96<br>(SPECIFY)<br>DON'T KNOW .... 88<br>REFUSED 99	
508	In the past 2 years has any visitor come to talk with you about preventing pregnancy or terminating an unintended pregnancy?	YES .... 1<br>NO .... 2<br>DON'T KNOW .... 88<br>REFUSED 99	2, 88, 99 → 524
509	What organization(s) was/were the visitor(s) from?<br><br>SELECT ALL THAT APPLY	WILLOWS .... 1<br>GOVERNMENT .... 2<br>OTHER, SPECIFY .... 96<br>DON'T KNOW .... 88<br>REFUSED 99	

NO.	QUESTIONS AND FILTERS	WILLOWS	OTHERS
510	CHECK 509: WILLOWS IF 1 & OTHERS IF 2 OR 96<br><br>In the last 2 years, how many times did someone come to visit to provide counseling or information?	1 TIME .... 1<br>(SKIP TO 512)<br>2 TIMES .... 2<br>3-5 TIMES .... 3<br>MORE THAN 5 TIMES .... 4<br>DON'T KNOW .... 88<br>REFUSED 99	1 TIME .... 1<br>(SKIP TO 512)<br>2 TIMES .... 2<br>3-5 TIMES .... 3<br>MORE THAN 5 TIMES ...... 4<br>DON'T KNOW .... 88<br>REFUSED 99
511	Did the same person come to visit you more than once?	YES .... 1<br>NO .... 2<br>DON'T KNO .... 88<br>REFUSED 99	YES .... 1<br>NO .... 2<br>DON' .... 88<br>REFU 99
512	What gender was the person(s) who came to visit?	MALE .... 1<br>FEMALE .... 2<br>BOTH MALE AND FEMALE VISITORS 88<br>REFUSED 99	MALE .... 1<br>FEMA .... 2<br>BOTH MALE AND FEMALE VIS 88<br>REFU 99
513	What topics did the person(s) talk with you about during the home visit?<br><br>SELECT ALL THAT APPLY	PREGNANCY 1<br>FAMILY PLANNING 2<br>ABORTION 3<br>SEXUALLY TRANSMITTED INFECTIONS (STIs) 4<br>PAP SMEAR (CERVICAL CANCER SCRI 5<br>BREAST EXAM 6<br>INFERTILITY 7<br>OTHER ______ 96<br>(SPECIFY)<br>DON'T KNOW 88<br>REFUSED 99	PREGNANCY 1<br>FAMILY PLANNING 2<br>ABORTION 3<br>SEXUALLY TRANSMITTED INFECTIONS 4<br>PAP SMEAR (CERVICAL CAN 5<br>BREAST EXAM 6<br>INFERTILITY 7<br>OTHER ______ 96<br>(SPECIFY)<br>DON'T KNOW 88<br>REFUSED 99
514	Were you counseled or provided information on using family planning methods?	YES .... 1<br>NO .... 2<br>DON'T KNOW 88<br>REFUSED 99<br>(SKIP TO 516)	YES .... 1<br>NO .... 2<br>DON'T KNOW 88<br>REFUSED 99<br>(SKIP TO 516)

5. FAMILY PLANNING

NO.	QUESTIONS AND FILTERS	CODING CATEGORIES		SKIP
515	Which method(s) were you counseled/given information on?<br><br>CIRCLE ALL MENTIONED.	FEMALE STERILIZATION 1<br>MALE STERILIZATION 2<br>IUD 3<br>INJECTABLES 4<br>IMPLANTS 5<br>PILL 6<br>CONDOM 7<br>FEMALE CONDOM 8<br>DIAPHRAGM 9<br>FOAM/JELLY 10<br>EMERGENCY CONTRACEPTION 11<br>STANDARD DAYS/ CALENDAR METHOI 12<br>LACTATIONAL AMEN. METH 13<br>RHYTHM METHOD 14<br>WITHDRAWAL 15<br>N TABLET 16<br>OTHER ________________ 17<br>NONE 18<br>DON'T KNOW/REMEMBER 88<br>REFUSED 99	FEMALE STERILIZA 1<br>MALE STERILIZATI(2<br>IUD 3<br>INJECTABLES 4<br>IMPLANTS 5<br>PILL 6<br>CONDOM 7<br>FEMALE CONDOM 8<br>DIAPHRAGM 9<br>FOAM/JELLY 10<br>EMERGENCY CONT 11<br>STANDARD DAYS/ CALENDAI 1212<br>LACTATIONAL AME 13<br>RHYTHM METHOD 14<br>WITHDRAWAL 15<br>N TABLET 1616<br>OTHER ________________ 17<br>NONE 18<br>DON'T KNOW/REMEMBER 88<br>REFUSED 99	
516	Who else (besides yourself) participated in the information sessions?<br><br>CIRCLE ALL MENTIONED.	HUSBAND/PARTNER 1<br>MOTHER 2<br>DAUGHTER 3<br>SON 4<br>FRIEND/NEIGHBOR 5<br>OTHERS 6<br>NO ONE ELSE 7<br>REFUSED 99	HUSBAND/PART 1<br>MOTHEF 2<br>DAUGHT 3<br>SON 4<br>FRIEND/NEIGHE 5<br>OTHERS 6<br>NO ONE ELSE 7<br>REFUSED 99	
517	Did she/he ever show you examples of contraceptive methods?	YES 1<br>NO 2<br>DON'T KNO 88<br>REFUSED 99	YES 1<br>NO 2<br>DON'T KNOW 88<br>REFUSED 99	
518	Did she/he ever give you brochures or materials about contraceptive methods?	YES 1<br>NO 2<br>DON'T KNO 88<br>REFUSED 99	YES 1<br>NO 2<br>DON'T KNOW 88<br>REFUSED 99	
519	Did she/he ever discuss induced abortion with you?	YES 1<br>NO 2<br>DON'T KNOW/ 88<br>REFUSED 99<br>(SKIP TO 521)	YES 1<br>NO 2<br>DON'T 88<br>REFUSED 99<br>(SKIP TO 521)	
520	Did she/he ever provide brochures or materials about induced abortion to you?	YES 1<br>NO 2<br>DON'T KNO 88<br>REFUSED 99	YES 1<br>NO 2<br>DON'T KNOW 88<br>REFUSED 99	
521	Did she/he ever refer you to a health facility for family planning or reproductive health issues?	YES 1<br>NO 2<br>DON'T KNO 88<br>REFUSED 99<br>(SKIP TO 523)	YES 1<br>NO 2<br>DON'T KNOW 88<br>REFUSED 99<br>(SKIP TO 523)	
522	Did you go there based on this referral?	YES 1<br>NO 2<br>DON'T KNO 88<br>REFUSED 99	YES 1<br>NO 2<br>DON'T KNOW 88<br>REFUSED 99	
523	Do you think the information provided by the field worker(s) was reliable?	YES 1<br>SOMEWHAT 2<br>NO 3<br>DON'T KNO 88<br>REFUSED 99	YES 1<br>SOMEWHAT 2<br>NO 3<br>DON'T KNOW 88<br>REFUSED 99	

5. FAMILY PLANNING

NO.	QUESTIONS AND FILTERS	CODING CATEGORIES	SKIP
524	In the past 2 years, have you visited a health facility for care for yourself (or your children)?	YES . . . 1<br>NO . . . 2<br>REFUSED . . . 99	1 → 526
525	Why have you not visited a health facility in the past 2 years? (Select all that apply)	TOO FAR . . . 1<br>TOO EXPENSIVE . . . 2<br>NO NEED . . . 3<br>OTHER, SPECIFY . . . 96<br>REFUSED . . . 99	→ 539
526	During any visit in the past 2 years, did any staff member at the health facility speak to you about family planning methods?	YES . . . 1<br>NO . . . 2<br>REFUSED . . . 99	2, 99 → 539
527	Which health facility did you receive family planning information from the last time?<br><br>PROBE TO IDENTIFY THE TYPE OF FACILITY.	**PUBLIC SECTOR**<br>GOVT. HOSPITAL/POLYCL . . . 11<br>GOVT. HEALTH CENTER/CLINIC . . . 12<br>GOVT. HEALTH POST/CHPS 13<br>FAMILY PLANNING CLINIC . . . 14<br>MOBILE CLINIC . . . 15<br>FIELDWORKER/OUTREACH/PEER EDUCATOR/ COMMUNITY HEALTH WORKER . . . 16<br>OTHER PUBLIC SECTOR ________ 17<br>(SPECIFY)<br><br>**PRIVATE MEDICAL SECTOR**<br>PRIVATE HOSPITAL/CLINIC . . . 18<br>PRIVATE DOCTOR . . . 19<br>PHARMACY . . . 20<br>CHEMICAL/DRUG STORE 21<br>FP/PPAG CLINIC 22<br>MATERNITY HOME 23<br>OTHER PRIVATE MEDICAL ________ 24<br>(SPECIFY)<br>OTHER ________ 96<br>(SPECIFY)<br><br>DON'T KNOW . . . 88<br>REFUSED . . . 99	
527B	What was this health facility called? (Name)	________	
528	Who provided you with this information or counseling (during the last visit)?<br><br>SELECT ALL THAT APPLY	DOCTOR . . . 1<br>NURSE . . . 2<br>MIDWIFE . . . 3<br>FIELDWORKER/OUTREA . . . 4<br>PEER EDUCATOR . . . 5<br>PHARMACIST<br>OTHER ________ 96<br>(SPECIFY)<br>DON'T KNOW . . . 88<br>REFUSED . . . 99	

5. FAMILY PLANNING			
NO.	QUESTIONS AND FILTERS	CODING CATEGORIES	SKIP
529	Which method(s) were you counseled on the last time?<br><br>CIRCLE ALL MENTIONED.	FEMALE STERILIZATION . . . . 1<br>MALE STERILIZATION . . . . 2<br>IUD . . . . 3<br>INJECTABLES . . . . 4<br>IMPLANTS . . . . 5<br>PILL . . . . 6<br>CONDOM . . . . 7<br>FEMALE CONDOM . . . . 8<br>DIAPHRAGM . . . . 9<br>FOAM/JELLY . . . . 10<br>EMERGENCY CONTRACEPTION . . . . 11<br>STANDARD DAYS/ CALENDAR METHOI . . . . 12<br>LACTATIONAL AMEN. METHOD . . . . 13<br>RHYTHM METHOD . . . . 14<br>WITHDRAWAL . . . . 15<br>N TABLET . . . . 16<br>OTHER ________________ . . . . 17<br>NONE . . . . 18<br>DON'T KNOW/REMEMBER 88<br>REFUSED 99	
530	**CHECK Q. 107:**<br><br>☐ CURRENTLY MARRIED/LIVING WITH PARTNER<br>*(Option 1)*	NOT CURRENLTY MARRIED OR LIVING TOGETHER WITH PARTNER ☐<br>*(Options 2,3,4)*	532
531	Did your husband/partner participate in the counseling/information session the last time?	YES . . . . 1<br>NO . . . . 2<br>REFUSED 99	
532	In which language(s) were you counseled the last time?<br><br>SELECT ALL THAT APPLY.	AKAN . . . . 1<br>GA . . . . 2<br>EWE . . . . 3<br>NZEMA . . . . 4<br>DAGBANI . . . . 5<br>ENGLISH . . . . 6<br>OTHER (specify) ______________________________ 96<br>DON'T REMEMBER/DON'T KNOW 88<br>REFUSED 99	
533	Did the person who counseled you ask questions about your health?	YES . . . . 1<br>NO . . . . 2<br>DON'T KNOW . . . . 88<br>REFUSED 99	
534	Were you counseled on what would happen if you chose NOT to use family planning?	YES . . . . 1<br>NO . . . . 2<br>DON'T KNOW . . . . 88<br>REFUSED 99	
535	Do you feel that you were given enough information about family planning?	YES . . . . 1<br>NO . . . . 2<br>DON'T KNOW . . . . 88<br>REFUSED 99	
536	Are you satisfied with the FAMILY PLANNING COUNSELING service you received at your last visit?	FULLY SATISFIED . . . . 1<br>SOMEWHAT SATISFIED . . . . 2<br>NOT SATISFIED . . . . 3<br>DON'T KNOW . . . . 88<br>REFUSED 99	
537	Do you think the family planning information provided by this service provider is reliable?	YES . . . . 1<br>SOMEWHAT . . . . 2<br>NO . . . . 3<br>DON'T KNOW . . . . 88<br>REFUSED 99	

5. FAMILY PLANNING

NO.	QUESTIONS AND FILTERS	CODING CATEGORIES	SKIP
538	Would you recommend services from this provider to a friend or a relative in a similar situation as you?	YES . . . . 1<br>NO . . . . 2<br>DON'T KNOW . . . . 88<br>REFUSED 99	
539	Have you <u>ever</u> used anything or tried in any way to delay or avoid getting pregnant?	YES . . . . 1<br>NO . . . . 2<br>REFUSED 99	(2, 99) → 541
540	Which method(s) have you ever used?<br><br>CIRCLE ALL MENTIONED.	FEMALE STERILIZATION . . . . 1<br>MALE STERILIZATION . . . . 2<br>IUD (Copper T, loop, wearing loop) 3<br>INJECTABLES (Depo-provera, N . . . . 4<br>IMPLANTS (Jadelle, Impla . . . . 5<br>PILL (Secure, Lydia, Microgynon, Microlut) 6<br>CONDOM . . . . 7<br>FEMALE CONDOM . . . . 8<br>DIAPHRAGM . . . . 9<br>FOAM/JELLY . . . . 10<br>EMERGENCY CONTRACEPTION (Postinor2, ... 11<br>STANDARD DAYS/ CALENDAR METHOI . . . . 12<br>LACTATIONAL AMEN. METHOD . . . . 13<br>RHYTHM METHOD . . . . 14<br>WITHDRAWAL . . . . 15<br>N TABLET . . . . 16<br>OTHER ________ . . . . 17<br>NONE . . . . 18<br>DON'T KNOW/REMEMBER 88<br>REFUSED 99	
541	In choosing a contraceptive method, what feature would be **most** important to you?<br><br>SELECT ONE-Probe for most important reason to the respondent	HOW EFFECTIVE IT IS AT PREVENTING PREGNANCY . . . . 1<br>CAN BE USED WITHOUT ANYONE ELSE KNOWING . . . . 2<br>THAT IT PROTECTS AGAINST STIs/HIV . . . . 3<br>NO RISK OF HARMING HEALTH 4<br>NO EFFECT ON REGULAR MONTHLY BLEEDING 5<br>NO UNPLEASANT SIDE EFFECTS 6<br>EASY TO USE 7<br>EASY TO OBTAIN 8<br>AFFORDABILITY 9<br>CAN BE USED FOR A LONG TIME WITHOUT NEED TO VISIT CLINIC OR RE-SUPPLY 10<br>WILL BE ABLE TO GET PREGNANT WHEN I WANT 11<br>OTHER ________ (SPECIFY) 96<br>DON'T KNOW . . . . 88<br>REFUSED 99	
542	CHECK Q. 539:<br><br>EVER USED FAMILY PLANNING ☐ ↓ NEVER USED FAMILY PLANNING ☐ →		548
543	CHECK Q. 420:<br><br>NOT PREGNANT OR UNSURE ☐ ↓ PREGNANT ☐ →		555
544	Are you or your husband/partner currently doing something or using any method to delay or avoid getting pregnant?	YES . . . . 1<br>NO . . . . 2<br>DON'T KNOW . . . . 88<br>REFUSED 99	(2, 88, 99) → 547

5. FAMILY PLANNING			
NO.	QUESTIONS AND FILTERS	CODING CATEGORIES	SKIP
545	Which method(s) are you or your husband/partner currently using?<br><br>RECORD ALL MENTIONED.<br><br>IF MORE THAN ONE METHOD MENTIONED, FOLLOW SKIP INSTRUCTION FOR HIGHEST METHOD IN LIST.	FEMALE STERILIZATION . . . . 1<br>MALE STERILIZATION . . . . 2<br>IUD (Copper T, loop, wearing loop) 3<br>INJECTABLES (Depo-provera, N . . . . 4<br>IMPLANTS (Jadelle, Impla . . . . 5<br>PILL (Secure, Lydia, Microgynon, Microlut) 6<br>CONDOM . . . . 7<br>FEMALE CONDOM . . . . 8<br>DIAPHRAGM . . . . 9<br>FOAM/JELLY . . . . 10<br>EMERGENCY CONTRACEPTION (Postinor2, ... 11<br>STANDARD DAYS/ CALENDAR METHOI . . . . 12<br>LACTATIONAL AMEN. METHOD . . . . 13<br>RHYTHM METHOD . . . . 14<br>WITHDRAWAL . . . . 15<br>N TABLET . . . . 16<br>OTHER ________________ . . . . 17<br>NONE . . . . 18<br>DON'T KNOW/REMEMBER 88<br>REFUSED 99	1, 2 → 556<br><br>88, 99 → 547
546	Since what month and year have you been using (CURRENT METHOD) without stopping?<br>INSERT 00 IF LESS THAN 1 MONTH.<br>PROBE: For how long have you been using (CURRENT METHOD) now without stopping?	MONTH . . . . [ ][ ]<br>YEAR . . . . [ ][ ][ ][ ]<br>DON'T KNOW . . . . 88/8888<br>REFUSED 99/9999	
547	Are you currently breastfeeding to delay or avoid getting pregnant?	YES . . . . 1<br>NO . . . . 2<br>DON'T KNOW . . . . 88<br>REFUSED 99	
548	CHECK Q. 424:<br>GAVE BIRTH IN PAST 6 MONTHS [ ] ↓<br>DID NOT GIVE GIVE BIRTH IN PAST 6 MONTHS [ ] →		553
549	Did you breastfeed your most recent baby at any time after giving birth?	YES . . . . 1<br>NO . . . . 2<br>REFUSED 99	2, 99 → 552
550	Are you still breastfeeding this baby?	YES . . . . 1<br>NO . . . . 2<br>REFUSED 99	2, 99 → 552
551	Have you given the baby anything but breastmilk since he/she was born?	YES . . . . 1<br>NO . . . . 2<br>REFUSED 99	
552	Has your period returned?	YES . . . . 1<br>NO . . . . 2<br>REFUSED 99	

5. FAMILY PLANNING

NO.	QUESTIONS AND FILTERS	CODING CATEGORIES	SKIP
553	CHECK Q. 539:<br>EVER USED FAMILY PLANNING ☐ ↓	NEVER USED FAMILY PLANNING ☐ →	579
554	CHECK Q. 544:<br>NOT CURRENTLY USING FAMILY PLANNING ☐ ↓	CURRENTLY USING FAMILY PLANNING ☐ →	556
555	You mentioned that you are not currently using a family planning method. Which method(s) did you use last?<br><br>RECORD ALL MENTIONED.<br><br>IF MORE THAN ONE METHOD MENTIONED, FOLLOW SKIP INSTRUCTION FOR HIGHEST METHOD IN LIST.	FEMALE STERILIZATION . . . . 1<br>MALE STERILIZATION . . . . 2<br>IUD . . . . 3<br>INJECTABLES . . . . 4<br>IMPLANTS . . . . 5<br>PILL . . . . 6<br>CONDOM . . . . 7<br>FEMALE CONDOM . . . . 8<br>DIAPHRAGM . . . . 9<br>FOAM/JELLY . . . . 10<br>EMERGENCY CONTRACEPTION . . . . 11<br>STANDARD DAYS/ CALENDAR METHOI . . . . 12<br>LACTATIONAL AMEN. METHOD . . . . 13<br>RHYTHM METHOD . . . . 14<br>WITHDRAWAL . . . . 15<br>N TABLET . . . . 16<br>OTHER ________________ . . . . 17<br>NONE . . . . 18<br>DON'T KNOW/REMEMBER 88<br>REFUSED 99	 <br><br><br><br><br><br><br><br><br><br><br><br><br><br><br><br><br>→ 579
556	Where did you obtain [THE LAST/CURRENT METHOD] the last time?<br><br>PROBE TO IDENTIFY THE TYPE OF SOURCE.<br><br>[Only for methods 1-11 in Question 545/555]	**PUBLIC SECTOR**<br>GOVT. HOSPITAL/POLYCL . . . . 11<br>GOVT. HEALTH CENTER/CLINIC . . . . 12<br>GOVT. HEALTH POST/CHPS 13<br>FAMILY PLANNING CLINIC . . . . 14<br>MOBILE CLINIC . . . . 15<br>FIELDWORKER/O . . . . 16<br>OTHER PUBLIC SECTOR ________________ 17<br>(SPECIFY)<br><br>**PRIVATE MEDICAL SECTOR**<br>PRIVATE HOSPITAL/CLINIC . . . . 18<br>PRIVATE DOCTOR . . . . 19<br>PHARMACY . . . . 20<br>CHEMICAL/DRUG STORE 21<br>FP/PPAG CLINIC 22<br>MATERNITY HOME 23<br>OTHER PRIVATE MEDICAL ________________ 24<br>(SPECIFY)<br>**OTHER SOURCE**<br>SHOP/MARKET 25<br>COMMUNITY VOLUNTEER 26<br>FRIEND/RELATIVE 27<br><br>OTHER ________________ 96<br>(SPECIFY)<br><br>DON'T KNOW . . . . 88<br>REFUSED 99	25–88 → 557
556 B	What was the name of the facility	NAME OF FACILITY ________________	
557	How many kilometers did you have to travel to reach this (SERVICE PROVIDER) to receive (LAST/CURRENT METHOD)?<br><br>[Only for methods 1-11 in Question 545/555]	KM . . . . ☐☐<br>DON'T KNOW 88<br>REFUSED 99	

5. FAMILY PLANNING			
NO.	QUESTIONS AND FILTERS	CODING CATEGORIES	SKIP
558	How many minutes did it take for you to travel to this (SERVICE PROVIDER) to receive [THE LAST/CURRENT METHOD]?<br><br>[Only for methods 1-11 in Question 545/555]	MINUTES . . . . [ ][ ][ ][ ]<br>REFUSED 99	
559	What mode(s) of transportation did you use to travel to this (SERVICE PROVIDER) to receive [THE LAST/CURRENT METHOD]?<br><br>[Only for methods 1-11 in Question 545/555]	NONE (RECEIVED AT HOME) 1<br>WALK 2<br>BICYCLE 3<br>MOTORCYCLE . . . . 4<br>BUS . . . . 5<br>CAR / TAXI 6<br>OTHER . . . . 96<br>REFUSED 99	
560	How much, in Ghanaian cedis, did you have to pay in total to obtain [THE LAST/CURRENT METHOD]?<br><br>IF NONE ENTER '00'<br><br>IF REFUSED ENTER '99'<br><br>[Only for methods 1-11 in Question 545/555]	COST OF METHOD [ ][ ][ ]<br>COST OF SERVICE [ ][ ][ ]<br>TRANSPORTATION [ ][ ][ ]<br>LOST WAGES [ ][ ][ ]<br>CHILDCARE . . [ ][ ][ ]	
561	How many minutes did you have to wait at the (SERVICE PROVIDER) before you received [THE LAST/CURRENT METHOD]?<br>[Only for methods 1-11 in Question 545/555]<br>IF REFUSED ENTER '9999'	MINUTES . . . . [ ][ ][ ][ ]	
562	Did you feel that you got enough information about [THE LAST/CURRENT METHOD]?<br>[Only for methods 1-11 in Question 545/555]	YES . . . . 1<br>NO . . . . 2<br>DON'T KNOW . . . . 88<br>REFUSED 99	
563	Did you receive any counseling or information from a health or family planning worker about [THE LAST/CURRENT METHOD]?<br>[Only for methods 1-11 in Question 545/555]	YES . . . . 1<br>NO . . . . 2<br>DON'T KNOW . . . . 88<br>REFUSED 99	NO → 572
564	Did the person who counseled you explain how [THE LAST/CURRENT METHOD] works to prevent pregnancy?<br>[Only for methods 1-11 in Question 545/555]	YES . . . . 1<br>NO . . . . 2<br>DON'T KNOW . . . . 88<br>REFUSED 99	
565	Did the person who counseled you on [THE LAST/CURRENT METHOD] explain how to use it?<br><br>[Only for methods 1-11 in Question 545/555]	YES . . . . 1<br>NO . . . . 2<br>DON'T KNOW . . . . 88<br>REFUSED 99	
566	Did the person who counseled you allow you to ask questions?<br><br>[Only for methods 1-11 in Question 545/555]	YES . . . . 1<br>NO . . . . 2<br>DON'T KNOW . . . . 88<br>REFUSED 99	
567	When you got [THE LAST/CURRENT METHOD], were you told by a health or family planning worker about other methods of family planning that you could use?<br><br>[Only for methods 1-11 in Question 545/555]	YES . . . . 1<br>NO . . . . 2<br>DON'T KNOW . . . . 88<br>REFUSED 99	

5. FAMILY PLANNING			
NO.	QUESTIONS AND FILTERS	CODING CATEGORIES	SKIP
568	When you got [THE LAST/CURRENT METHOD], were you told by a health or family planning worker about potential side effects or problems you might have with the method(s)?<br><br>[Only for methods 1-11 in Question 545/555]	YES . . . . 1<br>NO . . . . 2<br>DON'T KNOW . . . . 88<br>REFUSED 99	
569	When you got [THE LAST/CURRENT METHOD], were you told what to do if you experienced side effects or problems?<br><br>[Only for methods 1-11 in Question 545/555]	YES . . . . 1<br>NO . . . . 2<br>DON'T KNOW . . . . 88<br>REFUSED 99	
570	When you got [THE LAST/CURRENT METHOD], were you told about any benefits?<br><br>[Only for methods 1-11 in Question 545/555]	YES . . . . 1<br>NO . . . . 2<br>DON'T KNOW . . . . 88<br>REFUSED 99	
571	When you got [THE LAST/CURRENT METHOD], were you told of any disadvantages?<br><br>[Only for methods 1-11 in Question 545/555]	YES . . . . 1<br>NO . . . . 2<br>DON'T KNOW . . . . 88<br>REFUSED 99	
576	Was the method you wanted available to you?<br><br>[Only for methods 1-11 in Question 545/555]	YES . . . . 1<br>NO . . . . 2<br>DON'T KNOW . . . . 88<br>REFUSED 99	1 → 585

5. FAMILY PLANNING			
NO.	QUESTIONS AND FILTERS	CODING CATEGORIES	SKIP
577	What method had you wanted during that visit?<br><br><br>RECORD ALL MENTIONED.	FEMALE STERILIZATION . . . . . 1<br>MALE STERILIZATION . . . . . 2<br>IUD . . . . . 3<br>INJECTABLES . . . . . 4<br>IMPLANTS . . . . . 5<br>PILL . . . . . 6<br>CONDOM . . . . . 7<br>FEMALE CONDOM . . . . . 8<br>DIAPHRAGM . . . . . 9<br>FOAM/JELLY . . . . . 10<br>EMERGENCY CONTRACEPTION . . . . . 11<br>STANDARD DAYS/ CALENDAR METHOI . . . . . 12<br>LACTATIONAL AMEN. METHOD . . . . . 13<br>RHYTHM METHOD . . . . . 14<br>WITHDRAWAL . . . . . 15<br>N TABLET . . . . . 16<br>OTHER ________________ . . . . . 17<br>NONE . . . . . 18<br>DON'T KNOW/REMEMBER 88<br>REFUSED 99	
578	What was the MOST important reason didn't you obtain the method you wanted?	METHOD OUT OF STOCK THAT DAY 1<br>METHOD NOT AVAILABLE AT ALL 2<br>PROVIDER NOT TRAINED TO PROVIDE METHOD 3<br>PROVIDER RECOMMENDED ANOTHER METHOD 4<br>NOT ELIGIBLE FOR METHOD 5<br>DECIDED NOT TO ADOPT A METHOD 6<br>DECIDED TO ADOPT ANOTHER METHOD 7<br>TOO COSTLY 8<br>HUSBAND/PARTNER REFUSED 9<br>OTHER, SPECIFY ____________________________ 11<br>DON'T KNOW 88<br>REFUSED 99	
572	Did you feel pressured into using [THE LAST/CURRENT METHOD]?<br>[Only for methods 1-11 in Question 545/555]	YES . . . . . 1<br>NO . . . . . 2<br>DON'T KNOW . . . . . 88<br>REFUSED 99	
573	Did you feel you could say no to [THE LAST/CURRENT METHOD]?<br><br>[Only for methods 1-11 in Question 545/555]	YES . . . . . 1<br>NO . . . . . 2<br>DON'T KNOW . . . . . 88<br>REFUSED 99	
574	Has the [THE LAST/CURRENT METHOD] caused any problems?<br><br>[Only for methods 1-11 in Question 545/555]	YES . . . . . 1<br>NO . . . . . 2<br>DON'T KNOW . . . . . 88<br>REFUSED 99	
575	Do you feel that you have access to a wide range of family planning methods, or only to a select few?<br><br>[Only for methods 1-11 in Question 545/555]	A WIDE RANGE . . . . . 1<br>A SELECT FEW . . . . . 2<br>DON'T KNOW . . . . . 88<br>REFUSED 99	
579	CHECK Q. 539:<br><br>NEVER USED FAMILY PLANNING ☐ ↓	EVER USED FAMILY PLANNING ☐ →	585
580	Do you feel that you have access to a wide range of family planning methods?<br><br>READ OPTIONS ALOUD	A WIDE RANGE . . . . . 1<br>A SELECT FEW . . . . . 2<br>NO ACCESS . . . . . 3<br>DON'T KNOW . . . . . 88<br>REFUSED 99	
581	Have you ever wanted to use a method of family planning?	YES . . . . . 1<br>NO . . . . . 2<br>DON'T KNOW . . . . . 88<br>REFUSED 99	2, 88, 99 → 585
582	Is the method of family planning you would want to use available to you?	YES . . . . . 1<br>NO . . . . . 2<br>DON'T KNOW . . . . . 88<br>REFUSED 99	

5. FAMILY PLANNING			
NO.	QUESTIONS AND FILTERS	CODING CATEGORIES	SKIP
583	What method do you want or have you wanted most recently?<br><br>RECORD ALL MENTIONED.	FEMALE STERILIZATION . . . . . 1<br>MALE STERILIZATION . . . . . 2<br>IUD . . . . . 3<br>INJECTABLES . . . . . 4<br>IMPLANTS . . . . . 5<br>PILL . . . . . 6<br>CONDOM . . . . . 7<br>FEMALE CONDOM . . . . . 8<br>DIAPHRAGM . . . . . 9<br>FOAM/JELLY . . . . . 10<br>EMERGENCY CONTRACEPTION . . . . . 11<br>STANDARD DAYS/ CALENDAR METHOI . . . . . 12<br>LACTATIONAL AMEN. METHOD . . . . . 13<br>RHYTHM METHOD . . . . . 14<br>WITHDRAWAL . . . . . 15<br>N TABLET . . . . . 16<br>OTHER ________________ . . . . . 17<br>NONE . . . . . 18<br>DON'T KNOW/REMEMBER 88<br>REFUSED 99	
584	What is the MOST important reason you have not obtained the method you wanted?	METHOD OUT OF STOCK 1<br>METHOD NOT AVAILABLE AT ALL 2<br>PROVIDER NOT TRAINED TO PROVIDE METHOD 3<br>PROVIDER RECOMMENDED ANOTHER METHOD 4<br>NOT ELIGIBLE FOR METHOD 5<br>DECIDED NOT TO ADOPT A METHOD 6<br>DECIDED TO ADOPT ANOTHER METHOD 7<br>TOO COSTLY 8<br>HUSBAND/PARTNER REFUSED 9<br>DIFFICULT TO ACCESS HEALTH CENTER 10<br>OTHER, SPECIFY ____________________________ 11<br>DON'T KNOW 88<br>REFUSED 99	
	**CHECK IF MARRIED OR LIVING TOGETHER AS IF MARRIED=YES**		
585	Does your husband/partner support you in your choices related to contraceptive use?	YES . . . . . 1<br>NO . . . . . 2<br>DON'T KNOW . . . . . 88<br>REFUSED 99	
586	CHECK:<br><br>ANY BIRTH OR PREGNANCY TERMINATION AFTER MONTH AND YEAR OF START OF USE OF CONTRACEPTION IN Q. 546<br><br>GO BACK TO **546** PROBE AND RECORD MONTH AND YEAR AT START OF CONTINUOUS USE OF CURRENT METHOD (MUST BE AFTER LAST BIRTH OR PREGNANCY TERMINATION).	YES ☐ NO ☐	

5. FAMILY PLANNING

NO.	QUESTIONS AND FILTERS	CODING CATEGORIES	SKIP
587	CHECK Q. 546<br><br>JULY 2015 **(6)** OR LATER → C ENTER CODE FOR METHOD USED IN MONTH OF INTERVIEW IN THE CALENDAR AND IN EACH MONTH BACK TO THE DATE STARTED USING.<br><br>YEAR IS 2015 **(7)** OR EARLIER → C ENTER CODE FOR METHOD USED IN MONTH OF INTERVIEW IN THE CALENDAR AND EACH MONTH BACK TO JULY 2015 **(6)**.		
588	I would like to ask you some questions about the times you or your husband/partner may have used a method to avoid getting pregnant during the last 30 months.<br><br>USE CALENDAR TO PROBE FOR EARLIER PERIODS OF USE AND NONUSE, STARTING WITH MOST RECENT USE, BACK TO JULY 2015. **(6)**<br>USE NAMES OF CHILDREN, DATES OF BIRTH, AND PERIODS OF PREGNANCY AS REFERENCE POINTS.<br><br>C **IN COLUMN 1**, ENTER METHOD USE CODE OR '0' FOR NONUSE IN EACH BLANK MONTH.<br><br>ILLUSTRATIVE QUESTIONS:<br>* When was the last time you used a method? Which method was that?<br>* When did you start using that method? How long after the birth of (NAME)?<br>* How long did you use the method then?<br><br>**IN COLUMN 2**, ENTER CODES FOR DISCONTINUATION NEXT TO THE LAST MONTH OF USE. NUMBER OF CODES IN COLUMN 2 MUST BE SAME AS NUMBER OF INTERRUPTIONS OF METHOD USE IN COLUMN 1.<br><br>ASK WHY SHE STOPPED USING THE METHOD. IF A PREGNANCY FOLLOWED, ASK WHETHER SHE BECAME PREGNANT UNINTENTIONALLY WHILE USING THE METHOD OR DELIBERATELY STOPPED TO GET PREGNANT.<br><br>ILLUSTRATIVE QUESTIONS:<br>* Why did you stop using the (METHOD)? Did you become pregnant while using (METHOD), or did you stop to get pregnant, or did you stop for some other reason?<br>* IF DELIBERATELY STOPPED TO BECOME PREGNANT, ASK: How many months did it take you to get pregnant after you stopped using (METHOD)? AND ENTER ‘0’ IN EACH SUCH MONTH IN COLUMN 1.		

6. PREGNANCY

601	REVIEW THE CONTRACEPTIVE CALENDAR<br>IF THE WOMAN HAD A BIRTH IN THE PAST 30 MONTHS, ASK HER THE QUESTIONS ABOUT THIS LAST BIRTH. IF NOT SKIP THIS SECTION.<br><br>ONE OR MORE BIRTHS IN THE LAST 30 MONTHS ☐ ↓<br>NO BIRTHS IN THE LAST 30 MONTHS ☐ → 701<br><br>Now I would like to ask some questions about your most recent birth.	
602	When you got pregnant with this last baby, did you want to get pregnant at that time?	YES . . . . 1<br>(SKIP TO 605)<br>NO 2
603	Did you want to have a baby later on, or did you not want any children?	LATER . . . . 1<br>NO MORE/NONE . . . . 2<br>(SKIP TO 605)
604	How much longer did you want to wait?<br><br>RECORD IN UNIT RESPONDENT USES	MONTHS 1 ☐☐<br>YEARS 2 ☐☐<br>DON'T KNOW 88
605	Did you see anyone for antenatal care for this pregnancy?	YES . . . . 1<br>NO . . . . 2<br>(SKIP TO 611)
606	Whom did you see?<br><br>Anyone else?<br><br>PROBE TO IDENTIFY EACH TYPE OF PERSON AND RECORD ALL MENTIONED.	**HEALTH PERSONNEL**<br>DOCTOR . . . 1<br>NURSE/MIDWIFE 2<br>COM. HEALTH OFFICER/ NURSE . . . . . 3<br><br>**OTHER PERSON**<br>TRAD. BIRTH ATTENDANT (TBA) 4<br>VILLAGE HEALTH VOLUNTEER 5<br>TRAD. HEALTH PRACTITIONER 6<br><br>OTHER 7<br>(SPECIFY)<br><br>NO ONE ASSISTED 10<br>DON'T KNOW 88<br>REFUSED 99

607	Where did you receive antenatal care for this pregnancy?<br><br>Anywhere else?<br><br>PROBE TO IDENTIFY THE TYPE OF SOURCE.<br><br>IF UNABLE TO DETERMINE IF PUBLIC OR PRIVATE SECTOR, WRITE THE NAME OF THE PLACE.<br><br>______________________________<br>(NAME OF PLACE)	**HOME**<br>RESPONDENT'S HOME 1<br>OTHER HOME 2<br><br>**PUBLIC SECTOR**<br>GOV'T HOSPITAL 3<br>GOV'T HEALTH CENTER/CLINIC 4<br>GOV'T HEALTH POST/CHPS 5<br>MOBILE CLINIC 6<br>OTHER PUBLIC SECTOR<br>______________ 7<br>(SPECIFY)<br><br>**PRIVATE MEDICAL SECTOR**<br>PVT. HOSPITAL/ CLINIC 8<br>FP/PPAG CLINIC 9<br>MOBILE CLINIC 10<br>MATERNITY HOME 11<br>OTHER PRIVATE MED. SECTOR<br>______________ 12<br>(SPECIFY)<br><br>OTHER ______________ 96<br>(SPECIFY)<br>REFUSED 99
G_6_1	Did you have a maternal health book (Pink Book) for this pregnancy?<br><br>*If yes, ask to see it and use pink book to confirm responses below*	YES . . . . 1<br>NO . . . . 2<br>REFUSED 99
608	How many months pregnant were you when you first received antenatal care for this pregnancy?	MONTHS [|]<br><br>OBSERVED 1<br>REPORTED 2<br>DON'T KNOW 88<br>REFUSED 99
609	How many times did you receive antenatal care during this pregnancy (at facility or visit at home by provider)?	NUMBER OF TIMES [|]<br><br>OBSERVED 1<br>REPORTED 2<br>DON'T KNOW 88<br>REFUSED 99
610	Did you ever receive counseling on family planning during your antenatal care?	YES . . . . 1<br>NO . . . . 2<br>REFUSED 99

611	Did anyone assist with the delivery of this baby? Who assisted?<br><br>Anyone else?<br><br>PROBE FOR THE TYPE(S) OF PERSONS(S) AND RECORD ALL MENTIONED.<br><br>IF RESPONDENT SAYS NO ONE ASSISTED, PROBE TO DETERMINE WHETHER ANY ADULTS WERE PRESENT AT THE DELIVERY.	**HEALTH PERSONNEL**<br>DOCTOR . . . 1<br>NURSE/MIDWIFE 2<br>COM. HEALTH OFFICER/ NURSE . . . . . 3<br><br>**OTHER PERSON**<br>TRAD. BIRTH ATTENDANT/ TBA 4<br>VILLAGE HEALTH VOLUNTEER 5<br>TRAD. HEALTH PRACTITIONER 6<br>RELATIVE/NEIGHBOUR/FRIEND 7<br>OTHER ______ 8<br>(SPECIFY)<br><br>NO ONE ASSISTED 10<br>DON'T KNOW 88<br>REFUSED 99
612	Where did you give birth?<br><br>PROBE FOR THE TYPE OF SOURCE.<br><br>IF UNABLE TO DETERMINE IF PUBLIC OR PRIVATE SECTOR, WRITE THE NAME OF THE PLACE.<br><br>______<br>(NAME OF PLACE)	**HOME**<br>RESPONDENT'S HOME 11<br>OTHER HOME 12<br><br>**PUBLIC SECTOR**<br>GOV'T HOSPITAL 21<br>GOV'T HEALTH CENTER/CLINIC 22<br>GOV'T HEALTH POST CHPS 23<br>MOBILE CLINIC 24<br>OTHER PUBLIC SECTOR ______ 26<br>(SPECIFY)<br><br>**PRIVATE MEDICAL SECTOR**<br>PVT. HOSPITAL/ CLINIC 31<br>FP/PPAG CLINIC 32<br>MOBILE CLINIC 33<br>MATERNITY HOME 34<br>OTHER PRIVATE MED SECTOR ______ 36<br>(SPECIFY)<br><br>**OTHER** 96<br>______<br>(SPECIFY)<br>REFUSE 99

613	Did you have a post-natal care visit for this child at any time in the six weeks after the birth?	YES . . . . 1<br>NO . . . . 2<br>NO, BABY DIED……. 3<br>DON'T KNOW . . . . . 88<br>REFUSE 99
614	Did you receive family planning counseling at any post-natal visit?	YES . . . . 1<br>NO . . . . 2<br>DON'T KNOW . . . . . 88<br>REFUSE 99

7. MARRIAGE			
NO.	QUESTIONS AND FILTERS	CODING CATEGORIES	SKIP
701	CHECK 107:<br>EVER MARRIED/ PARTNER ☐ ↓<br>NEVER MARRIED/ LIVED WITH A MAN ☐ → 801		801
GS_7_1	Does your (husband/partner) have other wives or does he live with other women as if married?	YES . . . . 1<br>NO . . . . 2<br>DON'T KNOW . . . . 8<br>REFUSED 99	2, 8, 99 → 703
GS_7_2	Including yourself, in total, how many wives or live-in partners does he have?	TOTAL NUMBER OF WIVES AND LIVE-IN PARTNERS . . . ☐☐<br>DON'T KNOW . . . . 98<br>REFUSED 99	
GS_7_3	Are you the first, second, … wife?<br>IF REFUSED, ENTER '99'	RANK . . . . ☐☐	
706	Does your husband/partner currently live with you?	YES . . . . 1<br>NO . . . . 2<br>REFUSED 99	1 → 703<br>99 → 703
707	How long has your husband/partner been away?<br>RECORD IN UNIT RESPONDENT USES	MONTHS . . . . ☐☐<br>WEEKS . . . . ☐☐<br>YEARS ☐☐<br>DON'T KNOW 88<br>REFUSED 99	
703	Have you been married or lived with a man only once or more than once?	ONLY ONCE . . . . 1<br>MORE THAN ONCE . . . . 2<br>REFUSED 99	
704	CHECK 703:<br>MARRIED/ LIVED WITH A MAN ONLY ONCE ☐ ↓<br>In what month and year did you start living with your (husband/partner)?<br>MARRIED/ LIVED WITH A MAN MORE THAN ONCE ☐ ↓<br>Now I would like to ask about your first (husband/partner). In what month and year did you start living with him?	MONTH . . . . ☐☐<br>DON'T KNOW MONTH . . . . 98<br>REFUSED . . . . 99<br>YEAR . . . . ☐☐☐☐<br>DON'T KNOW YEAR . . . . 8888<br>REFUSED 9999	
705	How old were you when you first started living with him?<br>IF REFUSED ENTER '99'	AGE . . . . ☐☐	

SECTION 8. FERTILITY PREFERENCES & CONTRACEPTION

NO.	QUESTIONS AND FILTERS	CODING CATEGORIES	SKIP
801	CHECK 545:<br>NEITHER HE OR SHE STERILIZED ☐↓	HE OR SHE STERILIZED ☐	→ SECTION 9
802	CHECK 420:<br>PREGNANT ☐↓	NOT PREGNANT OR UNSURE ☐	→ 804
803	Now I have some questions about the future. After the child you are expecting now, would you like to have another child, or would you prefer not to have any more children?	HAVE ANOTHER CHILD . . . . 1<br>NO MORE . . . . 2<br>UNDECIDED/DON'T KNOW . . . . 88<br>REFUSED 99	1 → 805<br>2, 88, 99 → 811
804	Now I have some questions about the future. Would you like to have (a/another) child, or would you prefer not to have any (more) children?	HAVE (A/ANOTHER) CHILD . . . . 1<br>NO MORE/NONE . . . . 2<br>SAYS SHE CAN'T GET PREGNANT . . . . 3<br>UNDECIDED/DON'T KNOW . . . . '88<br>REFUSED 99	2 → 807<br>3 → 812<br>88 → 810
805	CHECK 420:<br>NOT PREGNANT OR UNSURE ☐↓<br>a) How long would you like to wait from now before the birth of (a/another) child?<br>PREGNANT ☐↓<br>b) After the birth of the child you are expecting now, how long would you like to wait before the birth of another child?	MONTHS . . . . 1 [ ][ ]<br>YEARS . . . . 2 [ ][ ]<br>SOON/NOW . . . . 93<br>SAYS SHE CAN'T GET PREGNANT . . . . 94<br>AFTER MARRIAGE . . . . 95<br>OTHER ______________ (SPECIFY) 96<br>DON'T KNOW . . . . 98<br>REFUSED 99	93 → 810<br>94 → 812<br>95, 96, 98, 99 → 810
806	CHECK 420:<br>NOT PREGNANT OR UNSURE ☐↓	PREGNANT ☐	→ 811
807	CHECK 544: USING A CONTRACEPTIVE METHOD?<br>NOT CURRENTLY USING ☐↓	CURRENTLY USING ☐	→ 812
808	CHECK 805:<br>'24' OR MORE MONTHS INTEND TO SPACE OR '02' OR MORE YEARS ☐↓<br>NOT ASKED ☐↓	'00-23' MONTHS INTEND TO LIMIT OR '00-01' YEAR ☐	→ 811

SECTION 8. FERTILITY PREFERENCES & CONTRACEPTION

NO.	QUESTIONS AND FILTERS	CODING CATEGORIES	SKIP
809	CHECK 804:<br>WANTS TO HAVE A/ANOTHER CHILD ☐ / WANTS NO MORE/ NONE ☐<br>a) You have said that you do not want (a/another) child soon. Can you tell me why you are not using a method to prevent pregnancy?<br>Any other reason?<br>b) You have said that you do not want any (more) children. Can you tell me why you are not using a method to prevent pregnancy?<br>Any other reason?<br>RECORD ALL REASONS MENTIONED.	NOT MARRIED . . . . . 1<br>**FERTILITY-RELATED REASONS**<br>NOT HAVING SEX . . . . . 2<br>INFREQUENT SEX . . . . . 3<br>MENOPAUSAL/HYSTERECTOMY . . . . . 4<br>CAN'T GET PREGNANT . . . . . 5<br>NOT MENSTRUATED SINCE LAST BIRTH . . . . . 6<br>BREASTFEEDING . . . . . 7<br>UP TO GOD/FATALISTIC . . . . . 8<br>**OPPOSITION TO USE**<br>RESPONDENT OPPOSED . . . . . 9<br>HUSBAND/PARTNER OPPOSED . . . . . 10<br>OTHERS OPPOSED . . . . . 11<br>RELIGIOUS PROHIBITION . . . . . 12<br>STIGMATIZING TO USE IN COMMUNITY .... 13<br>**LACK OF KNOWLEDGE**<br>KNOWS NO METHOD . . . . . 14<br>KNOWS NO SOURCE . . . . . 15<br>**METHOD-RELATED REASONS**<br>SIDE EFFECTS/HEALTH CONCERNS . . . . . 16<br>LACK OF ACCESS/TOO FAR . . . . . 17<br>METHOD COSTS TOO MUCH . . . . . 18<br>VISIT TO CLINIC COSTS TOO MUCH 19<br>CLINIC OPERATING HOURS LIMITED 20<br>NO DOCTOR OR PROVIDER AVAILABLE 21<br>PREFERRED METHOD NOT AVAILABLE . . . . . 22<br>NO METHOD AVAILABLE . . . . . 23<br>INCONVENIENT TO USE . . . . . 24<br>INTERFERES WITH BODY'S NORMAL PROCESSES . . . . . 25<br>OTHER ______ (SPECIFY) 96<br>DON'T KNOW . . . . . 88<br>REFUSED 99	
810	CHECK 544: USING A CONTRACEPTIVE METHOD?<br>NOT ASKED ☐ NO, NOT CURRENTLY USING ☐ YES, CURRENTLY USING ☐ → 812		812
811	Do you think you will use a contraceptive method to delay or avoid pregnancy at any time in the future?	YES . . . . . 1<br>NO . . . . . 2<br>DON'T KNOW . . . . . 88<br>REFUSED 99	
812	CHECK 410 & 412:<br>HAS LIVING CHILDREN ☐ / NO LIVING CHILDREN ☐<br>a) If you could go back to the time you did not have any children and could choose exactly the number of children to have in your whole life, how many would that be?<br>b) If you could choose exactly the number of children to have in your whole life, how many would that be?<br>PROBE FOR A NUMERIC RESPONSE.	NONE . . . . . 0<br>NUMBER . . . . . [ ][ ]<br>______<br>DON'T KNOW . . . . . 88<br>REFUSED 99	0 → 814<br>88 → 814<br>99 → 814

SECTION 8. FERTILITY PREFERENCES & CONTRACEPTION

NO.	QUESTIONS AND FILTERS	CODING CATEGORIES	SKIP
813	How many of these children would you like to be boys, how many would you like to be girls and for how many would it not matter if it's a boy or a girl?	BOYS / GIRLS / EITHER<br>NUMBER . . [ ][ ] [ ][ ] [ ][ ]<br>__________<br>DON'T KNOW . . . . 88<br>REFUSED 99	
814	CHECK 544: USING A CONTRACEPTIVE METHOD?<br>CHECK MARRIAGE: IN A MARRIAGE OR PARTNERSHIP?<br>CURRENTLY USING AND MARRIED/PARTNER [ ] ↓<br>NOT CURRENTLY AND MARRIED/PARTNER [ ] → 817<br>NOT MARRIED/IN PARTNERSHIP → 820		→ 817<br>→ 820
815	Does your husband/partner know that you are using a method of family planning?	YES . . . . 1<br>NO . . . . 2<br>DON'T KNOW . . . . 88<br>REFUSED 99	2, 88, 99 → 818
816	Now I have some questions about your current situation. Would you say that using contraception is mainly your decision, mainly your (husband's/partner's) decision, or did you both decide together?	RESPONDENT . . . . 1<br>HUSBAND/PARTNER . . . . 2<br>RESPONDENT & H . . . . 3<br>OTHER __________ 96<br>(SPECIFY)<br>DON'T KNOW . . . . 88<br>REFUSED 99	→ 818
817	Now I have some questions about your current situation. Would you say that **NOT** using contraception is mainly your decision, mainly your (husband's/partner's) decision, or did you both decide together?	RESPONDENT . . . . 1<br>HUSBAND/PARTNER . . . . 2<br>RESPONDENT & H . . . . 3<br>OTHER __________ 96<br>(SPECIFY)<br>DON'T KNOW . . . . 88<br>REFUSED 99	
818	Does your (husband/partner) want the same number of children that you want, or does he want more or fewer than you want?	SAME NUMBER . . . . 1<br>MORE CHILDREN . . . . 2<br>FEWER CHILDREN . . . . 3<br>DON'T KNOW . . . . 88<br>REFUSED 99	
819	If you were to not use/since you are not using any family planning method, how likely do you think it is that you will become pregnant during the next year?	LIKELY . . . . 1<br>UNSURE/NEUTRAL . . . . 2<br>UNLIKELY . . . . 3<br>REFUSED 99	
820	CHECK 544: USING A CONTRACEPTIVE METHOD?<br>CURRENTLY USING [ ] ↓<br>NOT CURRENTLY USING [ ] → 825		→ 825
821	If you were to continue to use your family planning method, how likely do you think it is that you would become pregnant during the next year?	LIKELY . . . . 1<br>UNSURE/NEUTRAL . . . . 2<br>UNLIKELY . . . . 3<br>REFUSED 99	
822	Different methods of family planning vary in how effective or ineffective they are in preventing pregnancy. How effective do you think that your family planning method is in preventing pregnancy? READ OPTIONS OUT LOUD	EFFECTIVE . . . . 1<br>SOMEWHAT EFFECTIVE . . . . 2<br>UNSURE/NEUTRAL . . . . 3<br>SOMEWHAT INEFFECTIVE . . . . 4<br>INEFFECTIVE . . . . 5<br>REFUSED 99	

SECTION 8. FERTILITY PREFERENCES & CONTRACEPTION

NO.	QUESTIONS AND FILTERS	CODING CATEGORIES	SKIP
823	Different methods of family planning vary in how convenient they are to use. How convenient is it to use your family planning method?<br><br>READ OPTIONS OUT LOUD	CONVENIENT . . . . 1<br>SOMEWHAT CONV. . . . . 2<br>UNSURE/NEUTRAL . . . . 3<br>SOMEWHAT INCOI . . . . 4<br>INCONVENIENT . . . . 5<br>REFUSEI 99	
824	Women may experience side-effects or discomfort while using a family planning method. How often do you experience any side-effect(s) or discomfort in use of your family planning method?	NEVER . . . . 1<br>SOMETIMES . . . . 2<br>OFTEN . . . . 3<br>ALL THE TIME . . . . 4<br>DON'T KNOW . . . . 88<br>REFUSEI 99	
825	CHECK 420:<br>NOT PREGNANT OR UNSURE ☐ ↓	PREGNANT ☐ →	830
826	If you were to get pregnant within the next year, would you look forward to telling your husband/partner? [ONLY ASK IF MARRIED]	YES . . . . 1<br>NO . . . . 2<br>DON'T KNOW . . . . 88<br>REFUSEI 99	
827	If you were to get pregnant within the next year, would you look forward to telling your parents you were pregnant?	YES . . . . 1<br>NO . . . . 2<br>PARENTS DECEASED 3<br>DON'T KNOW . . . . 88<br>REFUSEI 99	
828	If you were to get pregnant within the next year, would you be worried about how to financially support the child?	YES . . . . 1<br>NO . . . . 2<br>DON'T KNOW . . . . 88<br>REFUSEI 99	
829	If you were to get pregnant within the next year, would you consider terminating the pregnancy?	YES . . . . 1<br>NO . . . . 2<br>DON'T KNOW . . . . 88<br>REFUSEI 99	Skip to section 9
830	When you got pregnant with this pregnancy, did you look forward to telling your husband/partner? [ONLY ASK IF MARRIED]	YES . . . . 1<br>NO . . . . 2<br>DON'T KNOW . . . . 88<br>REFUSEI 99	
831	When you got pregnant with this pregnancy,, did you look forward to telling your parents you were pregnant?	YES . . . . 1<br>NO . . . . 2<br>PARENTS DECEASED 3<br>DON'T KNOW . . . . 88<br>REFUSEI 99	
832	When you got pregnant with this pregnancy, were you worried about how to financially support the child?	YES . . . . 1<br>NO . . . . 2<br>DON'T KNOW . . . . 88<br>REFUSEI 99	
833	When you got pregnant with this pregnancy, did you consider terminating the pregnancy?	YES . . . . 1<br>NO . . . . 2<br>DON'T KNOW . . . . 88<br>REFUSEI 99	

9. HUSBAND'S BACKGROUND AND WOMEN'S STATUS

NO.	QUESTIONS AND FILTERS	CODING CATEGORIES	SKIP
901	CHECK 107:<br>CURRENTLY MARRIED/ LIVING WITH A MAN ☐ ↓<br>FORMERLY MARRIED/ LIVED WITH A MAN ☐ → 903<br>NEVER MARRIED AND NEVER LIVED WITH A MAN ☐ → 904		903<br>904
902	How old was your (husband/partner) on his last birthday?	AGE IN COMPLETED YEARS [ ][ ]	
902B	Can your (husband/partner) read a phrase/sentence in English?	YES .... 1<br>NO .... 2<br>DON'T KNOW .... 88<br>REFUSED 99	
903	CHECK 107:<br>CURRENTLY MARRIED/ LIVING WITH A MAN ☐ ↓<br>What is your husband's/partner's occupation?<br>That is, what kind of work does he mainly do?<br>FORMERLY MARRIED/ LIVED WITH A MAN ☐ ↓<br>What was your (last) husband's/ partner's occupation?<br>That is, what kind of work did he mainly do?	AGRICULTURE AND LIVE .... 1<br>DAILY WAGE LABOURER .... 2<br>CIVIL SERVANT .... 3<br>OTHER PRIVATE SERVICE HOLD ..... 4<br>BUSINESS/ PETTY BUSIN .... 5<br>STUDENT .... 6<br>HOUSE WIFE ...... 7<br>OTHER ________________ 88<br>(SPECIFY)<br>REFUSED 99	
904	Aside from your own house chores, have you done any work in the last seven days?	YES .... 1<br>NO .... 2<br>REFUSED 99	1 → 908
905	As you know, some women take up jobs for which they are paid in cash or kind. Others sell things, have a small business or work on the family farm or in the family business.<br>In the last seven days, have you done any of these things or any other work, aside from household chores?	YES .... 1<br>NO .... 2<br>REFUSED 99	1 → 908
906	Although you did not work in the last seven days, do you have any job or business from which you were absent for leave, illness, vacation, maternity leave, or any other such reasons?	YES .... 1<br>NO .... 2<br>REFUSED 99	
907	Have you done any work in the last 12 months?	YES .... 1<br>NO .... 2<br>REFUSED 99	2, 99 → 909
908	What is your occupation, that is, what kind of work do you mainly do?	AGRICULTURE AND LIVE .... 1<br>DAILY WAGE LABOURER .... 2<br>CIVIL SERVANT .... 3<br>OTHER PRIVATE SERVICE HOLD ..... 4<br>BUSINESS/ PETTY BUSIN .... 6<br>STUDENT .... 7<br>HOUSE WIFE ...... 8<br>OTHER ________________ 88<br>(SPECIFY)<br>REFUSED 99	

909	CHECK 107:<br><br>CURRENTLY MARRIED/LIVING WITH A MAN ASI F MARRIED [ ] ↓ NOT MARRIED OR LIVING WITH MAN AS IF MARRIED [ ] →		919
910	Who usually decides how the money **you** earn will be used: mainly you, mainly your husband/partner, or you and your husband/partner jointly?<br><br>[ONLY IF 908 NOT SKIPPED]	RESPONDENT . . . . 1<br>HUSBAND/PARTNER . . . . 2<br>RESPONDEN' . . . . 3<br><br>OTHER ______________________ 96<br>(SPECIFY)<br>DON'T KNC . . . . 88<br>REFUS 99	
911	Would **you** say that the money that you earn is more than what your husband/partner earns, less than what he earns, or about the same?<br><br>[ONLY IF 908 NOT SKIPPED]	MORE THAN HIM . . . . 1<br>LESS THAN HIM . . . . 2<br>ABOUT THE SAME . . . . 3<br>HUSBAND/PARTNER DOESN'T<br>BRING IN ANY MONEY . . . . 4<br>DON'T KNOW . . . . 8<br>REFUSED 99	
912	Who usually decides how **your (husband's/partner's)** earnings will be used: you, your husband/partner, or you and your husband/partner jointly?	RESPONDENT . . . . 1<br>HUSBAND/PARTNER . . . . 2<br>RESPONDENT AND<br>HUSBAND/PARTNER JOINTLY . . . . 3<br>HUSBAND/PARTNER HAS<br>NO EARNINGS . . . . 4<br>OTHER ______________________ 6<br>(SPECIFY)<br>REFUSED 99	
913	Do you feel comfortable expressing your opinions to your husband/partner on working outside the home?	YES . . . . 1<br>NO . . . . 2<br>DON'T KNC . . . . 88<br>REFUSED 99	
914	Do you feel comfortable expressing your opinions to your husband/partner on going out unescorted?	YES . . . . 1<br>NO . . . . 2<br>DON'T KNC . . . . 88<br>REFUSED 99	
915	Do you feel comfortable expressing your opinions to your husband/partner on the education and care of your children?	YES . . . . 1<br>NO . . . . 2<br>DON'T KNC . . . . 88<br>REFUSED 99	

916	Who usually makes decisions about health care for yourself: you, your husband/partner, you and your husband/partner both, jointly, another member of your household, or someone else?	RESPONDENT = 1<br>HUSBAND/PARTNER = 2<br>RESPONDENT & HUSBAND/PARTNER JOINTLY = 3<br>OTHER MALE = 4<br>OTHER FEMALE = 6<br>88=DON'T KNOW<br>99=REFUSED<br><br>1 2 3 4 5 6 88 99	
917	Who usually makes decisions about making major household purchases?	CODES AS ABOVE<br><br>1 2 3 4 5 6 88 99	
918	Who usually makes decisions about visits to your family or relatives?	CODES AS ABOVE<br>1 2 3 4 5 6 88 99<br>→	
919	Do you own this or any other house either alone or jointly with someone else?	ALONE ONLY . . . . 1<br>JOINTLY ONLY . . . . 2<br>BOTH ALONE AND JOINTLY . . . . 3<br>DOES NOT OWN . . . . 4<br>REFUSEI 99	
920	Do you own any land either alone or jointly with someone else?	ALONE ONLY . . . . 1<br>JOINTLY ONLY . . . . 2<br>BOTH ALONE AND JOINTLY . . . . 3<br>DOES NOT OWN . . . . 4<br>REFUSED 99	
922	In your opinion, is a husband/partner justified in hitting or beating his wife in any of the following situations:<br><br>If she goes out without telling him?<br>If she neglects the children?<br>If she argues with him?<br>If she refuses to have sex with him?<br>If she burns the food?	YES NO DK R<br><br>1 GOES OUT . . . . 1 2 88 99<br>2 NEGL. CHILDREN . . . . 1 2 88 99<br>3 ARGUES . . . . 1 2 88 99<br>4 REFUSES SEX . . . . 1 2 88 99<br>5 BURNS FOOD 1 2 88 99	
923	Have you heard of these illnesses?<br>(1)HIV/AIDS<br>(2)Hepatitis B<br>(3)Syphilis<br>(4)Dysentery<br>(5)Gonorrhea<br>(6)Genital herpes<br>(7)Genital warts	YES NO M DK R<br>1 2 3 88 99<br>1 2 3 88 99<br>1 2 3 88 99<br>1 2 3 88 99<br>1 2 3 88 99<br>1 2 3 88 99<br>1 2 3 88 99	
924	Do you think that you have enough information about these types of illnesses?	YES............................ 1<br>NO .... 2<br>DON'T KNOW .... 88<br>REFUSE.................... 99	

925	Apart from AIDS, have you heard about other infections that can be transmitted through sexual contact?	YES........................... 1<br>NO .... 2<br>DON'T KNOW/UNSURE .... 88<br>REFUSE...................... 99	
926	Now I would like to ask you some questions about your health in the last 12 months. During the last 12 months, have you had a disease which you got through sexual contact?	YES........................... 1<br>NO .... 2<br>DON'T KNOW .... 88<br>REFUSE...................... 99	
927	Sometimes women experience a bad-smelling abnormal genital discharge. During the last 12 months, have you had a bad-smelling abnormal genital discharge?	YES........................... 1<br>NO .... 2<br>DON'T KNOW .... 88<br>REFUSE...................... 99	
928	Sometimes women have small, red or flesh colored bumps and blisters in the private area. During the last 12 months, have you ever had these?	YES........................... 1<br>NO .... 2<br>DON'T KNOW .... 88<br>REFUSE...................... 99	
929	If YES ON ANY OF 926/927/928, ASK:<br><br>The last time you that condition, did you seek any kind of advice or treatment?	YES........................... 1<br>NO .... 2<br>DON'T KNOW .... 88<br>REFUSE...................... 99	NO/DON'T KNOW/REFUSE → 1001
930	Where did you go?<br>Any other place?<br><br>PROBE TO IDENTIFY EACH TYPE OF SOURCE.<br>IF UNABLE TO DETERMINE IF PUBLIC OR PRIVATE (NAME OF PLACE(S))	**PUBLIC SECTOR**<br>GOVT. HOSPITAL/P . . . . 11<br>GOVT. HEALTH CENTER/CLI .... 12<br>GOVT. HEALTH POST/CHPS 13<br>FAMILY PLANNING CLI .... 14<br>MOBILE CLIN . . . . 15<br>FIELDWORKE . . . . 16<br>OTHER PUBLIC SECTOR __________ 17<br>(SPECIFY)<br><br>**PRIVATE MEDICAL SECTOR**<br>PRIVATE HOSPITAL/CLINIC . . . . 18<br>PRIVATE DOCTOR . . . . . . 19<br>PHARMACY . . . . 20<br>CHEMICAL/DRUG STORE 21<br>FP/PPAG CLINIC 22<br>MATERNITY HOME 23<br>OTHER PRIVATE MEDICAL __________ 24<br>(SPECIFY)<br>**OTHER SOURCE**<br>SHOP/MARKET 25<br>COMMUNITY VOLUNTEER 26<br>FRIEND/RELATIVE 27<br><br>OTHER__________ 96<br>(SPECIFY)<br><br>DON'T KNO . . . . 88<br>REFUSEI 99	

10. OTHER HEALTH ISSUES

NO.	QUESTIONS AND FILTERS	CODING CATEGORIES	SKIP
1001	Many different factors can prevent women from getting family planning advice or treatment for themselves. When you want to get family planning advice or treatment, is each of the following a big problem or not?<br><br>Getting permission to go to the doctor?<br><br>Getting money needed for advice or treatment?<br><br>The distance to the health facility?<br><br>Not wanting to go alone?<br><br>Not having time because of work?<br><br>No one to look after children?	BIG PROB-LEM / NOT A BIG PROB-LEM / DK / R<br><br>1 PERMISSION TO GO . 1 2 # 99<br><br>2 GETTING MONEY .... 1 2 # 99<br><br>3 DISTANCE .... 1 2 # 99<br><br>4 GO ALONE .... 1 2 # 99<br><br>5 NO TIME DUE TO WORK 1 2 # 99<br><br>6 NO CHILDCARE 1 2 # 99	
1002	Are you covered by any health insurance?	YES .... 1<br>NO .... 2<br>REFUSED .... #	NO → 1005
1003	What type of health insurance are you covered by?<br>RECORD ALL MENTIONED.	NATIONAL/DISTRICT HEALTH INSURANCE (NHIS) .... 1<br>HEALTH INSURANCE THROUGH EMPLOYEF .... 2<br>MUTUAL HEALTH ORGANIZATION/ COMMUNITY-BASED HEALTH INSURANCE .... 3<br>OTHER PRIVATELY PURCHASED COMMERCIAL HEALTH INSURANCE 4<br>OTHER ______________ #<br>(SPECIFY)<br>DON'T KNC .... #<br>REFUSED .... #	
GH_10_1	Can I see your insurance card or other evidence of this insurance cover?	Yes, Card seen with current cover 1<br>Yes, Card seen but no current cover 2<br>No, Card not seen 3<br>REFUSED .... #	
1004	Does your insurance cover any of the following maternity benefits:<br><br>a) Antenatal health care?<br>b) Childbirth health care in a health facility?<br>c) Postnatal health care for the mother?<br>d) Postnatal health care for the child?<br>e) Cash benefits during maternity leave?<br>f) Family planning methods or services?<br>g) Abortion services?<br>h) Other?	YES / NO / DK / RF<br><br>1 ANTENATAL (ANC) 1 2 # 99<br>2 CHILDBIRTH 1 2 # 99<br>3 PNC MOTHER 1 2 # 99<br>4 PNC CHILD .... 1 2 # 99<br>5 CASH BENEFITS 1 2 # 99<br>6 FAMILY PLANNING 1 2 # 99<br>7 ABORTION 1 2 # 99<br>8 OTHER .... 1 2 # 99	
1005	RECORD THE TIME.	HOUR .... [ ][ ]<br><br>MINUTES .... [ ][ ]	

FOLLOW-UP INFORMATION

FOLLOW-UP INFORMATION

Thank you for this information. We are planning to conduct follow-up interviews with some women whom we are interviewing now. If you are selected for a follow-up interview, you will be contacted beforehand using the contact information that you have given us, and you will be provided with information on how to participate in the interview.

CONTACT 1

FULL NAME: ______________________________

RELATIONSHIP TO YOU: ______________________________

LOCATION OF HOUSE/DIRECTIONS: ______________________________

____

____

PHONE NUMBER: ______________________________

E-MAIL ADDRESS: ______________________________

CONTACT 2

FULL NAME: ______________________________

RELATIONSHIP TO YOU: ______________________________

LOCATION OF HOUSE/DIRECTIONS: ______________________________

____

____

PHONE NUMBER: ______________________________

E-MAIL ADDRESS: ______________________________

OTHER HOUSEHOLD CONTACT:

What is the name and phone number of someone else within your household?

NAME: ______________________________

PHONE: ______________________________

RELATIONSHIP TO YOU: ______________________________

PLANS TO MOVE:

Does your family/household have any plans to move in the next two years? YES NO

IF YES: Where do you plan to move to? LOCATION: ______________________________

____

____

CONTRACEPTIVE CALENDAR

INSTRUCTIONS:

ONLY ONE CODE SHOULD APPEAR IN ANY BOX.

COLUMN 1 REQUIRES A CODE IN EVERY MONTH.

INFORMATION TO BE CODED FOR EACH COLUMN

COLUMN 1: <u>BIRTHS, PREGNANCIES, CONTRACEPTIVE USE</u>**

B BIRTHS
P PREGNANCIES
T TERMINATIONS

0 NO METHOD
1 FEMALE STERILIZATION
2 MALE STERILIZATION
3 IUD
4 INJECTABLES
5 IMPLANTS
6 PILL
7 CONDOM
8 FEMALE CONDOM
9 EMERGENCY CONTRACEPTION
J RHYTHM METHOD/STANDARD DAYS METHOD/CYCLEBEADS
K LACTATIONAL AMENORRHEA METHOD
L N_TABLET
M WITHDRAWAL
X OTHER MODERN METHOD
Y OTHER TRADITIONAL METHOD

COLUMN 2: <u>DISCONTINUATION OF CONTRACEPTIVE USE</u>

0 INFREQUENT SEX/HUSBAND AWAY
1 BECAME PREGNANT WHILE USING
2 WANTED TO BECOME PREGNANT
3 HUSBAND/PARTNER DISAPPROVED
4 WANTED MORE EFFECTIVE METHOD
5 SIDE EFFECTS/HEALTH CONCERNS
6 LACK OF ACCESS/TOO FAR
7 COSTS TOO MUCH
8 INCONVENIENT TO USE
F UP TO GOD/FATALISTIC
A DIFFICULT TO GET PREGNANT/MENOPAUSAL
D MARITAL DISSOLUTION/SEPARATION
X OTHER ______________________________
(SPECIFY)
Z DON'T KNOW

* Year of fieldwork is assumed to be 2018. For fieldwork beginning in 2019 or 2020, the years should be adjusted.

** Response categories may be added for other methods, including fertility awareness methods.

				1	2	
	12	DEC	1			
	11	NOV	2			
	10	OCT	3			
	9	SEP	4			
2	8	AUG	5			2
0	7	JUL	6			0
1	6	JUN	7			1
8	5	MAY	8			8
*	4	APR	9			*
	3	MAR	10			
	2	FEB	11			
	1	JAN	12			
	12	DEC	13			
	11	NOV	14			
	10	OCT	15			
	9	SEP	16			
2	8	AUG	17			2
0	7	JUL	18			0
1	6	JUN	19			1
7	5	MAY	20			7
*	4	APR	21			*
	3	MAR	22			
	2	FEB	23			
	1	JAN	24			
	12	DEC	25			
	11	NOV	26			
	10	OCT	27			
	9	SEP	28			
2	8	AUG	29			2
0	7	JUL	30			0
1	6	JUN	31			1
6	5	MAY	32			6
*	4	APR	33			*
	3	MAR	34			
	2	FEB	35			
	1	JAN	36			
	12	DEC	37			
	11	NOV	38			
	10	OCT	39			
	9	SEP	40			
2	8	AUG	41			2
0	7	JUL	42			0
1	6	JUN	43			1
5	5	MAY	44			5
*	4	APR	45			*
	3	MAR	46			
	2	FEB	47			
	1	JAN	48			

END OF SURVEY:

You have now reached the end of the interview. Thank you for your time and responses. This has been extremely helpful. As I said in the beginning, the purpose of this discussion was to help us learn about women's health in Ghana I also want to remind you that all of your responses will remain confidential.

Do you have any further questions for me at this time?

Thank you again for your help.

SIGNATURE OF INTERVIEWER:____________________________________ DATE: ______________________

END. GO TO INTERVIEWER OBSERVATIONS.

INTERVIEWER'S OBSERVATIONS

TO BE FILLED IN AFTER COMPLETING INTERVIEW

COMMENTS ABOUT INTERVIEW:

COMMENTS ON SPECIFIC QUESTIONS:

ANY OTHER COMMENTS:

EDITOR'S OBSERVATIONS
